# The Application Status of Radiomics-Based Machine Learning in Intrahepatic Cholangiocarcinoma: Systematic Review and Meta-Analysis

**DOI:** 10.2196/69906

**Published:** 2025-05-05

**Authors:** Lan Xu, Zian Chen, Dan Zhu, Yingjun Wang

**Affiliations:** 1 Department of First Clinical Medical College Zhejiang Chinese Medical University Hangzhou China; 2 Dispensary TCM Quzhou Municipal Hospital of Traditional Chinese Medicine Quzhou China; 3 Department of Dermatology Quzhou Municipal Hospital of Traditional Chinese Medicine Quzhou China

**Keywords:** radiomic, machine learning, intrahepatic cholangiocarcinoma, bile duct cancer, systematic review, meta-analysis

## Abstract

**Background:**

Over the past few years, radiomics for the detection of intrahepatic cholangiocarcinoma (ICC) has been extensively studied. However, systematic evidence is lacking in the use of radiomics in this domain, which hinders its further development.

**Objective:**

To address this gap, our study delved into the status quo and application value of radiomics in ICC and aimed to offer evidence-based support to promote its systematic application in this field.

**Methods:**

PubMed, Web of Science, Cochrane Library, and Embase were comprehensively retrieved to determine relevant original studies. The study quality was appraised through the Radiomics Quality Score. In addition, subgroup analyses were undertaken according to datasets (training and validation sets), imaging sources, and model types.

**Results:**

Fifty-eight studies encompassing 12,903 patients were eligible, with an average Radiomics Quality Score of 9.21. Radiomics-based machine learning (ML) was mainly used to diagnose ICC (n=30), microvascular invasion (n=8), gene mutations (n=5), perineural invasion (PNI; n=2), lymph node (LN) positivity (n=2), and tertiary lymphoid structures (TLSs; n=2), and predict overall survival (n=6) and recurrence (n=9). The C-index, sensitivity (SEN), and specificity (SPC) of the ML model developed using clinical features (CFs) for ICC detection were 0.762 (95% CI 0.728-0.796), 0.72 (95% CI 0.66-0.77), and 0.72 (95% CI 0.66-0.78), respectively, in the validation dataset. In contrast, the C-index, SEN, and SPC of the radiomics-based ML model for detecting ICC were 0.853 (95% CI 0.824-0.882), 0.80 (95% CI 0.73-0.85), and 0.88 (95% CI 0.83-0.92), respectively. The C-index, SEN, and SPC of ML constructed using both radiomics and CFs for diagnosing ICC were 0.912 (95% CI 0.889-0.935), 0.77 (95% CI 0.72-0.81), and 0.90 (95% CI 0.86-0.92). The deep learning–based model that integrated both radiomics and CFs yielded a notably higher C-index of 0.924 (0.863-0.984) in the task of detecting ICC. Additional analyses showed that radiomics demonstrated promising accuracy in predicting overall survival and recurrence, as well as in diagnosing microvascular invasion, gene mutations, PNI, LN positivity, and TLSs.

**Conclusions:**

Radiomics-based ML demonstrates excellent accuracy in the clinical diagnosis of ICC. However, studies involving specific tasks, such as diagnosing PNI and TLSs, are still scarce. The limited research on deep learning has hindered both further analysis and the development of subgroup analyses across various models. Furthermore, challenges such as data heterogeneity and interpretability caused by segmentation and imaging parameter variations require further optimization and refinement. Future research should delve into the application of radiomics to enhance its clinical use. Its integration into clinical practice holds great promise for improving decision-making, boosting diagnostic and treatment accuracy, minimizing unnecessary tests, and optimizing health care resource usage.

## Introduction

Intrahepatic cholangiocarcinoma (ICC), as the second most frequent primary hepatic carcinoma, represents nearly 20% of hepatic malignant tumor cases [1]. Originating from cholangiocytes or progenitor cells [2], ICC is characterized by a poor prognosis, with a mortality-to-incidence ratio as high as 0.95 and a 5-year survival rate below 20% [3]. In most countries, the mortality rate of ICC is rising. The estimated annual percentage change in mortality is 6.9%±1.5% in males and 5.1%±1.0% in females [4]. Concurrently, the ICC incidence has grown by more than 140% over the past 40 years [5]. From 1973 to 2012, the incidence of ICC in America rose from 0.44 to 1.18 cases per 100,000 people, with an annual percentage change of 2.30% [6]. Given its escalating incidence and high fatality rate, ICC has garnered significant attention in recent years.

Early ICC diagnosis remains highly challenging as it is asymptomatic and goes unnoticed until reaching an advanced stage in most cases [7]. Therefore, early detection and diagnosis are crucial. However, the sensitivity (SEN) of cytological and pathological methods for early diagnosis of ICC is often limited due to factors such as subtle laboratory abnormalities and the presence of small tumors in anatomically challenging locations within the liver [6]. Complete surgical resection is the sole possible curative method for ICC, yet merely 20%-30% of patients are eligible for surgery [8]. Moreover, studies have highlighted that ICC is biologically aggressive and is prone to early vascular invasion, metastasis [9], and lymph node (LN) involvement, which are strongly linked to poor prognosis [10]. Pathological prognostic indicators include vascular invasion, periductal infiltration, tumor heterogeneity, local extension, and LN metastasis [2]. Timely diagnosis and treatment of ICC are imperative, so more scientific tools are needed.

Preoperative tumor staging and resectability assessments for ICC largely rely on imaging findings. Conventional imaging techniques, however, are heavily dependent on the expertise of radiologists, potentially leading to misdiagnosis. Radiomics, a burgeoning field in oncology, has been used in ICC. In recent years, the exponential growth of medical image analysis, thanks to expanding datasets and advanced pattern recognition tools, has facilitated the high-throughput extraction of quantitative features, such as image size, shape, and texture [11]. Initially put forward by Lambin et al [12] in 2012, radiomics can derive high-dimensional quantitative characteristics from biomedical images and transform imaging data into high-throughput, quantitative datasets for analysis. The process involves (1) image acquisition, (2) identification of prognostically relevant volumes, (3) segmentation of these volumes using computer-assisted contouring techniques, (4) extraction and qualitative assessment of descriptive features, (5) systematic compilation of these features into a searchable database, and (6) mining of the data for the development of predictive models, either independently or through integration with demographic, clinical, comorbidity, or genomic data. When combined with clinical data, it offers complementary information and enhances diagnostic accuracy [13]. Early diagnosis and prognostic prediction for ICC remain formidable challenges in clinical practice. Radiomics-based machine learning (ML) has been used to detect microvascular invasion (MVI) and predict recurrence. However, a systematic body of evidence validating the efficiency and status quo of radiomics-based ML is lacking, posing challenges to the development of radiomics in this domain. Therefore, our study seeks to review the accuracy of ML constructed by radiomics in the detection and prognostic prediction of ICC and offer evidence-based references to advance the intelligent diagnosis and treatment of this disease.

## Methods

### Study Registration

Our study followed the PRISMA (Preferred Reporting Items for Systematic Reviews and Meta-Analyses) statement [14]. The protocol has been registered in the International Prospective Register of Systematic Reviews (CRD42024583829). The PRISMA checklist is shown in Multimedia Appendix 1.

### Eligibility Criteria

The following studies were included: (1) those that developed complete ML models constructed by radiomics for the diagnosis and prognostic prediction of ICC; (2) original studies that conducted only internal validation without external validation; we also acknowledge the contributions of articles without external validation and have summarized the results of both the training and validation sets, which can help check for potential overfitting (therefore, these studies were also included); (3) English articles; and (4) case-control, cohort, or cross-sectional research.

The following reports were excluded: (1) meta-analyses, reviews, guidelines, expert opinions, or conference abstracts released with no peer review; (2) those that performed image segmentation without complete radiomics models for the diagnosis and prognostic prediction of ICC; and (3) those lacking one of outcome measures as follows for evaluating model accuracy: C-index, SEN, specificity (SPC), accuracy, precision, confusion matrix, diagnostic 4-fold table, or *F*_1_-score.

### Data Sources and Search Strategy

PubMed, Web of Science, Embase, and the Cochrane Library were retrieved from their inception until August 23, 2024. MeSH (Medical Subject Headings) and free-text terms were used, with no restraint on region or publication year. The strategy is shown in Table S1 in Multimedia Appendix 2.

### Study Selection and Data Extraction

Reviewers independently uploaded the searched records into EndNote and excluded duplicate entries. Subsequently, they reviewed all titles and abstracts, selected relevant original studies, downloaded full texts, and further screened them to identify the eligible studies.

A standard data extraction form was created for extracting specific information: the first author, publication year, country, design, patient source, outcome events, radiomics source, segmentation methods, imaging study participants, region of interest segmentation software, total case number, the number of cases in the validation or training group, validation set–generating method, and model type. In addition, outcome measures were extracted to assess model accuracy, including C-index, SEN, SPC, accuracy, precision, confusion matrix, and diagnostic 4-fold table. Literature screening and information extraction were separately implemented by 2 investigators (LX and ZC) and then cross-check was implemented. If any dissent arose, a third analyzer (YW) would address them.

### Assessment of Study Quality

The study quality is assessed via the Radiomics Quality Score and the Quality Assessment of Diagnostic Accuracy Studies 2 (QUADAS-2). The Radiomics Quality Score includes 16 items, with each study section corresponding to a specific project. Scores were assigned based on the methodological quality and summed to generate an overall score for each project. The range of total scores was from –8 to +36. Scores between –8 and 0 were considered 0%, and a score of 36 denoted 100% [15].

QUADAS-2 has been extensively applied in systematic reviews of diagnostic accuracy studies across various medical fields. The tool evaluates 4 domains in terms of risk of bias and concerns regarding applicability, with 3 possible categorizations: high risk, unclear risk, and low risk. The assessment of risk of bias specifically includes 4 key items: patient selection, the index test under evaluation, the reference standard’s implementation and interpretation, and patient flow. Each item comprises 3 specific assessment questions. If any of these questions are rated as high risk, the corresponding item is classified as high risk [16]. Two investigators (LX and ZC) appraised the quality of the eligible studies. Upon completion, they cross-checked the results. If any disagreements arose, a third researcher (YW) was consulted for the final decision.

### Outcomes

In this systematic review, the outcome measures were the area under the receiver operating characteristic curve (AUROC), SEN, and SPC, which reflected the accuracy of ML models constructed by radiomics.

### Synthesis Methods

A meta-analysis was conducted on the C-index, an indicator of the overall accuracy of ML models. When 95% CI and SE of the C-index were lacking, the SE was conjectured based on the method put forward by Debray et al [16]. The heterogeneity index (*I*²) was used to assess interstudy heterogeneity. When *I*² surpassed 50%, a random-effects model was used for analysis. Conversely, when *I*² was less than 50%, a fixed-effects model was applied.

Moreover, a bivariate mixed-effects model was leveraged to implement the meta-analysis of SEN and SPC according to diagnostic 4-fold tables. Nevertheless, most original studies offered no such tables. In such cases, the diagnostic 4-fold tables were derived based on SEN, SPC, precision, and case numbers. The meta-analysis was implemented leveraging Stata (version 15.0; StataCorp).

## Results

### Results of Literature Screening

**Figure 1:**
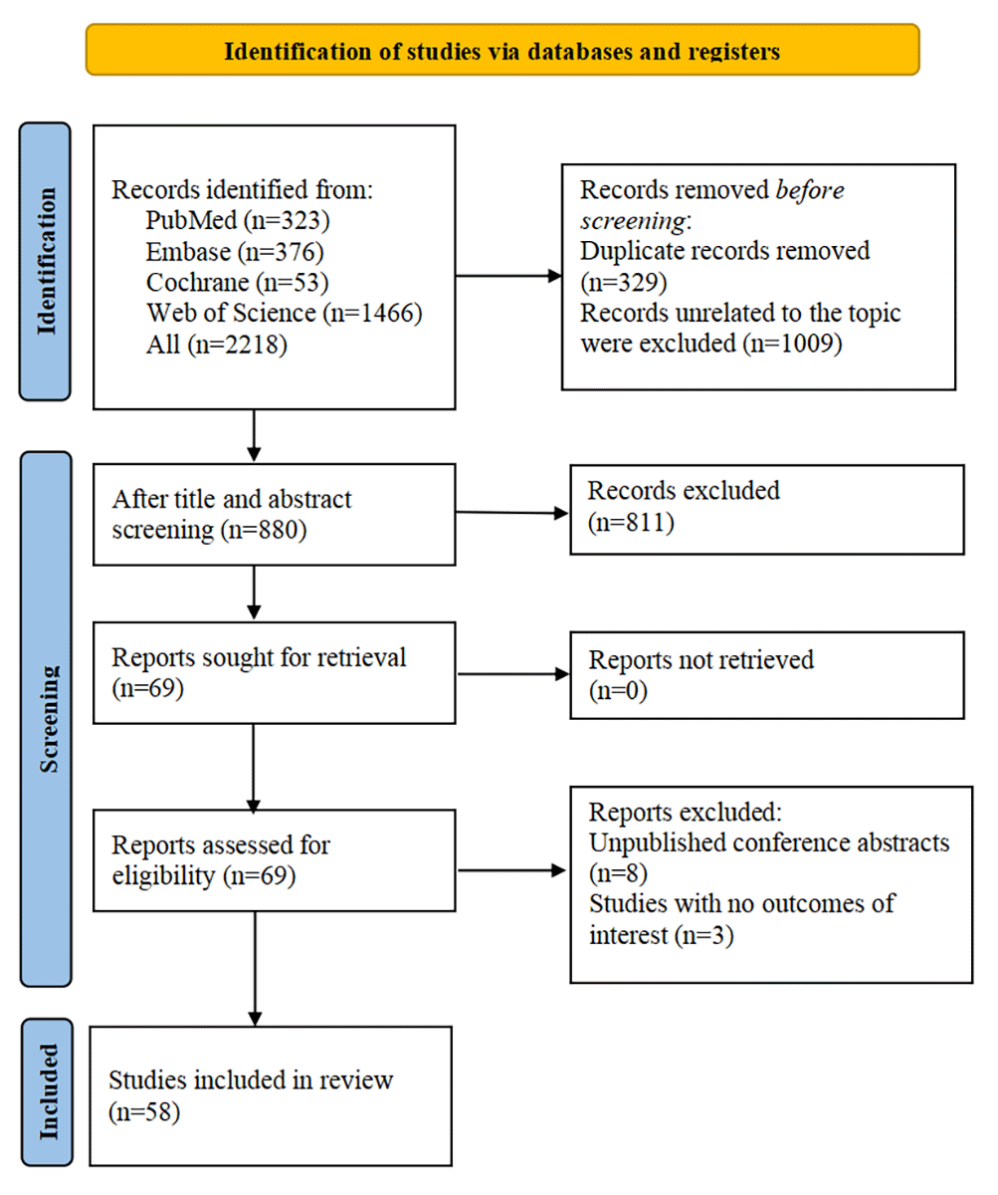
Literature screening process.

Initially, 2218 studies were identified, of which 329 were duplicates. Next, after 1009 records were removed for other reasons, we evaluated the titles and abstracts and found 69 potentially eligible reports. Subsequently, 3 studies with no outcomes of interest and 8 conference abstracts were removed. Eventually, 58 studies were included. Figure 1 shows the PRISMA flowchart.

### Basic Characteristics of Eligible Studies

Fifty-eight studies were included, comprising 15 cohort studies and 41 case-control studies, with 2 sourced from public databases. Of all studies, 40 were single-center studies, 8 were dual-center studies, and 10 were multicenter studies. Sample sizes ranged from 36 to 764 cases. These studies focused primarily on the diagnosis, risk stratification, and prognostic prediction of ICC.

Thirty eligible studies reported the diagnostic performance of radiomics-based ML for ICC; 6 studies evaluated overall survival (OS) in patients with ICC; 8 studies assessed MVI; 9 studies investigated recurrence; 5 studies analyzed gene mutations; 2 studies reported perineural invasion (PNI); 2 studies evaluated LN positivity; 2 studies assessed tertiary lymphoid structures (TLSs); 2 studies reported progression-free survival (PFS); 1 study examined noncurative resection; 3 studies assessed recurrence-free survival; 3 studies evaluated tumor grading; 1 study addressed radiographic response in patients with ICC undergoing transarterial radioembolization; and 1 study focused on vascular endothelial growth factor and cytokeratin 7.

Across all studies, 13 studies used deep learning (DL), while 45 used ML approaches. Among these, images were manually segmented in 44 studies, semiautomated segmentation was adopted in 5 studies, and automatic segmentation was leveraged in the remaining 9 DL-based studies. ITKSNAP (Penn Image Computing and Science Laboratory, University of Pennsylvania) and LifeX (IMIV or CEA, Université Paris-Saclay) were the most commonly used software tools for region of interest segmentation. A total of 47 studies carried out internal validation, while 12 studies implemented external validation. Most studies (n=29) assessed radiomics performance using computed tomography (CT), while approximately one-third (n=19) relied on magnetic resonance imaging (MRI). Moreover, 1 study delved into CT combined with MRI, while 2 studies leveraged positron emission tomography (PET) or CT. Finally, 7 studies used ultrasonography (US). The specific types of radiomics varied significantly across studies. In diagnostic models, logistic regression was mostly adopted, whereas Cox regression was primarily used in prognostic prediction models. Other algorithms, such as random forests, support vector machines, neural networks, and XGBoost, were also used. Since the number of incorporated studies was large, these algorithms were not elaborated in detail (Table S2 in Multimedia Appendix 2).

### Quality Assessment

**Figure 2:**
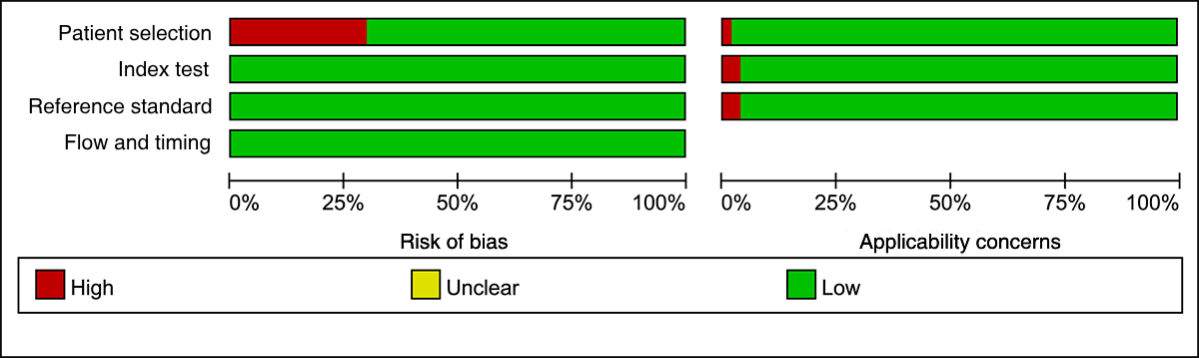
A cumulative bar plot of risk of bias and applicability concerns across all studies.

**Figure 3:**
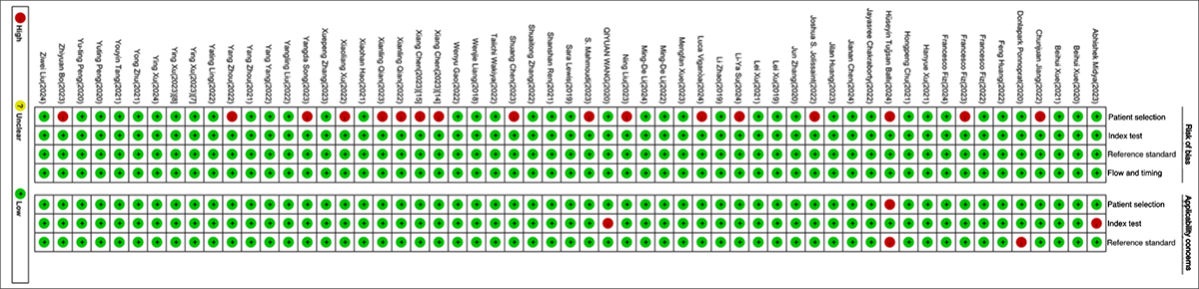
A summary table of review authors' ratings of risk of bias and applicability concerns for each study.

Among the 58 eligible studies, 9 studies had no explicit description of imaging protocols, resulting in a score of 0 for the Image Protocol Quality criterion. In 2 studies, segmentation was not conducted by different physicians, algorithms, or software, nor did they perform segmentation with (random) noise interference or across varying respiratory cycles, yielding no points for the multiple segmentation item. Twenty-two studies did not acquire images at different time points, leading to a score of 0 for this aspect. Forty-four studies incorporated multivariable analyses with nonradiomic features, such as Ki-67 mutation, which could potentially provide more comprehensive models; however, these studies did not evaluate or discuss biological relevance. Nineteen studies performed cutoff value analyses, reducing the risk of reporting overly optimistic results, earning 1 point for this criterion. Among all included studies, 40 reported discriminatory statistics and their statistical significance, while 18 additionally used resampling techniques, earning 1 and 2 points, respectively. For calibration statistics, 32 studies reported calibration and its statistical significance, and 2 applied resampling techniques. All eligible studies were not prospectively registered in a trial database, resulting in a score of 0 for this criterion. Four studies lacked validation, scoring –5 points. Thirty-eight studies conducted validation using datasets from the same institution, earning 2 points. Two studies performed validation using datasets from another institution, scoring 3 points. Nine studies performed validation using datasets from 2 different institutions, scoring 4 points, while 5 studies performed validation with datasets from 3 or more institutions, earning 5 points. Only 3 studies assessed the concordance of their models with current “gold standard” methods, earning points in this domain. Seventeen studies reported the current or possible clinical applications, whereas only 2 included cost-effectiveness analyses in a clinical setting. For open-access segmentation of the regions of interest, 53 studies scored 1 point, while 5 studies did not make any code or data publicly available. As a result, the scores of the included studies varied between –2 and 14, with a mean score of 9.21 (25.6%). Further details are shown in Table S3 in Multimedia Appendix 2. Details of the QUADAS-2 assessment are shown in Figures 2 and 3. Our assessment revealed that 17 studies exhibited a high risk of bias in the patient selection domain, while 4 studies demonstrated a high risk of bias regarding applicability concerns, as evaluated via the QUADAS-2 tool.

### Meta-Analysis Results

#### Diagnostic Tasks

##### Diagnosis of ICC

In the training group, the C-index, SEN, and SPC of ML models constructed by clinical features (CFs) were 0.814 (95% CI 0.760-0.869), 0.79 (95% CI 0.72-0.84), and 0.80 (95% CI 0.71-0.87), whereas those of models constructed based exclusively on radiomic characteristics were 0.876 (95% CI 0.851-0.901), 0.83 (95% CI 0.80-0.86), and 0.86 (95% CI 0.81-0.90). The models constructed by both radiomics and CFs achieved a C-index, SEN, and SPC of 0.940 (95% CI 0.922-0.959), 0.90 (95% CI 0.85-0.93), and 0.90 (95% CI 0.87-0.93).

As for the validation set, the CFs-based models yielded a C-index of 0.762 (95% CI 0.728-0.796), SEN of 0.72 (95% CI 0.66-0.77), and SPC of 0.72 (95% CI 0.66-0.78), while the C-index, SEN, and SPC of the models constructed based on only radiomic were 0.853 (95% CI 0.824-0.882), 0.80 (95% CI 0.73-0.85), and 0.88 (95% CI 0.83-0.92). The models constructed by both radiomics and CFs displayed a C-index of 0.912 (95% CI 0.889-0.935), SEN of 0.77 (95% CI 0.72-0.81), and SPC of 0.90 (95% CI 0.86-0.92). Given that different models and image sources may influence the diagnostic accuracy, subgroup analyses were performed. Detailed results of subgroup analyses can be found in Tables S4 and S5 in Multimedia Appendix 2.

##### Diagnosis of MVI

In the training cohort, the C-index, SEN, and SPC of the CFs-based models were 0.717 (95% CI 0.679-0.755), 0.55 (95% CI 0.38-0.72), and 0.79 (95% CI 0.66-0.88). For the models based on radiomics features, these values were 0.918 (95% CI 0.870-0.966), 0.83 (95% CI 0.68-0.91), and 0.90 (95% CI 0.64-0.98). In the models based on radiomics and CFs, they were 0.881 (95% CI 0.834-0.928), 0.80 (95% CI 0.75-0.84), and 0.83 (95% CI 0.75-0.89).

In the validation cohort, the models built on CFs yielded a C-index of 0.599 (95% CI 0.471-0.727), with SEN ranging from 0.1 to 0.58 and SPC ranging from 0.61 to 0.96. For the radiomics-based models, the C-index, SEN, and SPC were 0.851 (95% CI 0.808-0.894), 0.75 (95% CI 0.68-0.81), and 0.84 (95% CI 0.78-0.89). In the models based on both radiomics with CFs, the C-index, SEN, and SPC were 0.836 (95% CI 0.792-0.881), 0.77 (95% CI 0.67-0.84), and 0.88 (95% CI 0.79-0.94). Considering the potential influence of different model or image sources on accuracy, subgroup analysis was conducted. Tables S6 and S7 in Multimedia Appendix 2 show detailed results.

##### Diagnosis of Genetic Mutations

In the training group, the model developed as per CFs yielded a C-index of 0.677 (95% CI 0.543-0.812), with SEN and SPC ranging from 0.20 to 0.73 and 0.60 to 0.93. As for the models based on radiomics, the C-index, SEN, and SPC were 0.800 (95% CI 0.737-0.864), 0.85 (95% CI 0.69-0.93), and 0.91 (95% CI 0.86-0.94). In the models based on radiomics and CFs, C-index, SEN, and SPC were 0.892 (95% CI 0.822-0.962), 0.93 (95% CI 0.55-0.99), and 0.69 (95% CI 0.11-0.98).

As for the validation set, the CFs-based model had a C-index of 0.594 (95% CI 0.541-0.646), with a SEN of 0.98 (95% CI 0.82-1.00) and SPC of 0.02 (95% CI 0.00-0.31). In the models developed by radiomics, the C-index was 0.902 (95% CI 0.855-0.949), and SEN and SPC ranged from 0.909 to 0.911 and from 0.69 to 0.81. The C-index of the models developed by radiomics and CFs was 0.889 (95% CI 0.851-0.927), with SEN varying between 0.82 and 0.96 and SPC ranging from 0.33 to 0.76. Considering the potential influence of different model or image sources on accuracy, subgroup analysis was performed. Details are illustrated in Tables S8 and S9 in Multimedia Appendix 2.

##### Diagnosis of PNI

Concerning the training group, the C-index of models constructed using CFs was 0.731 (95% CI 0.598-0.864), with SEN and SPC ranging from 0.61 to 0.73, and from 0.74 to 0.79, respectively. In contrast, the models constructed by radiomics and CFs demonstrated a higher C-index of 0.890 (95% CI 0.817-0.964), with SEN from 0.84 to 0.85 and SPC from 0.73 to 0.98.

Regarding the validation cohort, the pooled C-index of the models constructed by CFs was 0.746 (95% CI 0.604-0.889), and SEN and SPC ranged from 0.43 to 1.00 and 0.32 to 1.00. The C-index of models developed by radiomics and CFs was 0.865 (95% CI 0.806-0.924), and SEN and SPC ranged from 0.67 to 0.86 and 0.79 to 0.95. Considering that different models and imaging sources may influence accuracy, subgroup analyses were performed. The detailed results are shown in Tables S10 and S11 in Multimedia Appendix 2.

##### Diagnosis of LN Positivity

Within the training set, the aggregated clinical features–based model exhibited 0.768 (95% CI 0.561-0.976) for C-index, with SEN and SPC ranging from 0.64 to 0.91 and 0.67 to 0.68. The C-index, SEN, and SPC of models developed by radiomics were 0.817 (95% CI 0.732-0.901), 0.68 (95% CI 0.55-0.78), and 0.86 (95% CI 0.79-0.91). The models developed by both radiomics and CFs showed a C-index of 0.916 (95% CI 0.781-1.000), and the SEN and SPC ranged from 0.87 to 0.89 and 0.58 to 0.97.

Within the validation cohort, the C-index, SEN, and SPC of models developed based on CFs were 0.768 (95% CI 0.682-0.853), 0.71-0.89, and 0.41-0.74. As for the models constructed using radiomics features, they were 0.769 (95% CI 0.726-0.813), 0.67 (95% CI 0.52-0.79), and 0.79 (95% CI 0.67-0.87). The C-index of models developed by both radiomics and CFs was 0.912 (95% CI 0.873-0.951), with SEN and SPC ranging from 0.79 to 0.89 and 0.70 to 0.90. Given the potential impact of varying models and imaging sources on accuracy, subgroup analyses were carried out. The specific results are detailed in Tables S12 and S13 in Multimedia Appendix 2.

##### Diagnosis of TLSs

Within the training cohort, the C-index, SEN, and SPC of CFs-based models were 0.750 (95% CI 0.630-0.870), 0.76, and 0.80. In the models based on radiomics, they were 0.754 (95% CI 0.592-0.915), 0.29-0.89, and 0.69-0.91. The C-index, SEN, and SPC of models based on both radiomics and CFs were 0.850 (95% CI 0.775-0.925), 0.57, and 0.93.

Within the validation group, the C-index, SEN, and SPC of models developed by CFs were 0.710 (95% CI 0.485-0.935), 0.80, and 0.76. As for the radiomics-based models, they were 0.735 (95% CI 0.589-0.881), 0.61 (95% CI 0.22-0.89), and 0.90 (95% CI 0.80-0.95). The C-index, SEN, and SPC of models developed by both radiomics and CFs were 0.880 (95% CI 0.760-1.000), 0.60, and 0.93. Considering that different models and image sources may influence accuracy, a subgroup analysis was carried out. The subgroup results are shown in Tables S14 and S15 in Multimedia Appendix 2.

##### Diagnosis of Tumor Grading

Among the encompassed studies, only 3 addressed tumor grading in patients with ICC, and only 1 study reported results from the validation cohort. Owing to limited studies, no meta-analysis was implemented. Fiz et al [17] reported that the DL model constructed by both radiomics and CFs achieved the highest C-index of 0.767 (95% CI 0.497-1.000), with SEN and SPC of 0.789 and 0.741 in the training set. Fiz et al [18] reported that the ML model developed based on both radiomics and CFs in the training group had the highest C-index of 0.834 (95% CI 0.738-0.930), with SEN and SPC of 0.652 and 0.787. Based on data from the validation cohort, Chen et al [19] noted that the ML model based on both radiomics and CFs achieved the highest C-index of 0.869 (95% CI 0.783-0.955), with SEN and SPC of 0.759 and 0.821. Additional studies are warranted to corroborate the use of radiomics for tumor grading.

#### Prediction Task

##### Prediction of OS

As for the training group, the pooled C-index of the CFs-based models was 0.726 (95% CI 0.662-0.790). SEN and SPC were not given in the included studies. The summary C-index for the models developed by integrating radiomics and CFs was 0.765 (95% CI 0.746-0.785), and SEN and SPC were 0.64 and 0.69. Within the validation group, the summary C-index for the CFs-based models was 0.670 (95% CI 0.565-0.775), and the other 2 indicators were not provided. In the model based on both radiomics and CFs, the C-index, SEN, and SPC were 0.763 (95% CI 0.657-0.869), 0.69, and 0.61. Considering that different models and image sources may impact accuracy, subgroup analyses were performed. Detailed results of the subgroup analyses are shown in Tables S16 and S17 in Multimedia Appendix 2.

##### Prediction of Recurrence

In the training group, the C-index, SEN, and SPC of the models constructed using CFs were 0.761 (95% CI 0.720-0.802), 0.72 (95% CI 0.64-0.79), and 0.65 (95% CI 0.57-0.72). For the models based on radiomics characteristics alone, the C-index, SEN, and SPC were 0.766 (95% CI 0.756-0.776), 0.78 (95% CI 0.75-0.82), and 0.86 (95% CI 0.81-0.90). The C-index, SEN, and SPC of models based on both radiomics and CFs were 0.837 (95% CI 0.775-0.898), 0.85 (95% CI 0.78-0.90), and 0.77 (95% CI 0.70-0.84).

Within the validation cohort, the C-index, SEN, and SPC of models developed by CFs were 0.727 (95% CI 0.681-0.773), 0.64 (95% CI 0.56-0.71), and 0.64 (95% CI 0.56-0.71). These values of radiomics-based models were 0.816 (95% CI 0.790-0.841), 0.84 (95% CI 0.78-0.88), and 0.76 (95% CI 0.69-0.82). The C-index, SEN, and SPC of models based on both radiomics and CFs were 0.863 (95% CI 0.810-0.917), 0.85 (95% CI 0.80-0.88), and 0.74 (95% CI 0.67-0.79). Given the potential influence of different models or image sources on the accuracy, a subgroup analysis was performed. The specific results are shown in Tables S18 and S19 in Multimedia Appendix 2.

##### Prediction of PFS

Among the included studies, only 2 explored the use of radiomics in predicting PFS. Because studies were insufficient, a meta-analysis was not conducted. Both studies reported only training set results, without SEN or SPC. Fiz et al [20] noted that the model developed by both radiomics and CFs displayed the highest C-index, at 0.750 (95% CI 0.698-0.802). Fiz et al [18] demonstrated that the C-index of models constructed by radiomics and CFs was the highest, at 0.860 (95% CI 0.702-1.000). The performance of radiomics needs to be further validated.

## Discussion

### Principal Findings

Our study uncovers that radiomics is extensively used in ICC, for instance, in the diagnosis of ICC, MVI, LN positivity, genetic mutations, PNI, TLSs, and prediction of recurrence. Among these, the primary focus is on diagnosing ICC, identifying genetic mutations, detecting MVI, and predicting recurrence, wherein radiomics exhibited relatively favorable accuracy. In the validation group, the C-index, SEN, and SPC of models constructed by CFs for detecting ICC were 0.762 (95% CI 0.728-0.796), 0.72 (95% CI 0.66-0.77), and 0.72 (95% CI 0.66-0.78). The C-index, SEN, and SPC of radiomic-based models were 0.853 (95% CI 0.824-0.882), 0.80 (95% CI 0.73-0.85), and 0.88 (95% CI 0.83-0.92). The C-index, SEN, and SPC of models based on both radiomics and CFs were 0.912 (95% CI 0.889-0.935), 0.77 (95% CI 0.72-0.81), and 0.90 (95% CI 0.86-0.92).

Previous studies have explored various diagnostic tools for ICC. A 2019 meta-analysis assessed the robustness of cytokeratin-19 fragment (CYFRA21-1) for diagnosing ICC and reported an AUROC of 0.904 (SE 0.0171), SEN of 0.81 (95% CI 0.75-0.86), and SPC of 0.86 (95% CI 0.82-0.89) [21]. A retrospective study investigating the prognostic effects of CT and MRI features on ICC included 204 patients who underwent curative ICC resection. It identified enhancement patterns and infiltrative tumor margins as predictors of OS and event-free survival, while biliary invasion was determined to be a predictor of OS [22]. You and Yun [23] identified 11 MRI characteristics for distinguishing hepatocellular carcinoma (HCC) from ICC. Among these, valuable MRI characteristics with high diagnostic odds ratios (DORs >20) included arterial rim enhancement, progressive enhancement, and a target appearance on hepatobiliary phase images. In addition, a meta-analysis [24] compared the performance of MRI and contrast-enhanced ultrasonography (CEUS) to differentiate ICC from HCC. For MRI, the SEN, SPC, DOR, and AUROC were reported as 0.81 (95% CI 0.79-0.84), 0.90 (95% CI 0.88-0.91), 41.47 (95% CI 24.07-71.44), and 0.93 (95% CI 0.90-0.96). For CEUS, the SEN, SPC, DOR, and AUROC were 0.88 (95% CI 0.84-0.90), 0.80 (95% CI 0.78-0.83), 42.06 (95% CI 12.38-133.23), and 0.93 (95% CI 0.87-0.99). Although CEUS demonstrated higher pooled SEN, MRI showed superior pooled SPC. However, comprehensive reviews addressing recurrence and MVI are scarce, with most research focusing on these aspects in HCC [25-27]. A meta-analysis on CT radiomics in anticipating MVI in HCC reported pooled SEN, SPC, and AUROC values of 0.82 (95% CI 0.77-0.86), 0.79 (95% CI 0.75-0.83), and 0.87 (95% CI 0.84-0.91) [25]. Radiomics-based ML demonstrated favorable performance in detecting MVI in ICC in our analysis. In the validation cohort, the C-index, SEN, and SPC of models developed by both radiomics and CFs were 0.836 (95% CI 0.792-0.881), 0.77 (95% CI 0.67-0.84), and 0.88 (95% CI 0.79-0.94). The models developed solely on radiomics or CFs should be further validated and used in clinical practice.

In existing studies, the primary imaging modalities include CT, PET-CT, MRI, and US. The NCCN Clinical Practice Guidelines [28] recommend the use of MRI and CT in combination with clinical evaluation for diagnosis. In actual clinical practice, MRI and CT are the most commonly used imaging techniques. Our study revealed that in the validation cohort, models constructed by MRI and CFs outperformed those based on CT or US combined with CFs in diagnosing ICC, with C-index values of 0.922 (95% CI 0.896-0.949), 0.896 (95% CI 0.853-0.939), and 0.902 (95% CI 0.835-0.969), respectively. MRI exhibited a highly superior diagnosis performance in this context. Furthermore, subgroup analyses based on alternative outcomes in our study yielded variable results. MRI, CT, and other modalities showed optimal efficacy depending on the specific outcome, as detailed in Tables S4-S19 in Multimedia Appendix 2. These findings suggest that the selection of imaging modalities can be tailored to specific diagnostic objectives without additional, unnecessary imaging modalities.

Image segmentation has emerged as a critical process in radiomics. Segmentation techniques can be broadly categorized into manual segmentation and DL-based automatic segmentation [29]. Among the studies included in our analysis, 45 used traditional ML approaches, of which only 2 used semiautomatic segmentation, while the remainder relied exclusively on manual segmentation. In DL-related studies, 1 used manual segmentation. Overall, current radiomics workflows are predominantly dependent on manual segmentation, which poses several challenges. First, the vector knowledge and reproducibility of the individuals performing manual segmentation require further evaluation. The segmentation variability arising from different operators performing manual segmentation possibly introduced uncertainty in the evaluation of radiomics models, thereby affecting the reproducibility of the model. In the future, the application of artificial intelligence for automatic segmentation or the adoption of more standardized segmentation protocols will mitigate the impact of manual segmentation. Second, the dependency on variations among different manufacturers of the same equipment and different imaging parameters for the same image should be assessed. However, the studies included in our analysis did not conduct preliminary experiments in this regard. Third, the impact of different imaging phases should be further discussed. Among the eligible studies, only a limited number of studies described images obtained at different phases, such as arterial, venous, delayed, or mixed phases. Since studies reporting such outcomes were insufficient, further analysis on this was not undertaken.

The models based on radiomics primarily consist of traditional ML and DL models [30]. In traditional ML, texture features need to be extracted from segmented regions, and features should be selected for model construction. However, the feature selection is challenging, primarily due to the complexity of diverse feature selection methodologies and the potential loss of raw image information. These factors may compromise the accuracy of models based on ML [31]. Moreover, in the case of a large number of features, it is complicated to eliminate the risk of overfitting during the modeling process [32]. In our study, the C-index between the 2 sets was relatively similar, which indicated that the risk of overfitting was effectively mitigated in the current research. In the context of DL, the processes of image feature extraction and texture selection are integrated into the learning process, thereby preserving the integrity of image information to a greater extent [33]. Our findings indicate that DL demonstrates superior performance in comparison with traditional ML. In the diagnosis of ICC, radiomics-based DL models markedly outperformed other models in the validation cohort. The C-index values of CT, MRI, and US were 0.937 (95% CI 0.893-0.982), 0.968 (95% CI 0.903-1.033), and 0.891 (95% CI 0.786-0.996), respectively. Furthermore, among models constructed by both radiomics and CFs, subgroup analysis based on US-derived images uncovered that the C-index of DL models was 0.924 (95% CI 0.863-0.984), notably higher than other approaches. However, these results are derived from a limited set of models, and further exploration in future studies is warranted to substantiate these findings.

In ML research, the validation methods of models have drawn significant attention. Most existing studies primarily adopted internal validation to assess models. In internal validation, the training data and validation data were derived from the same dataset, with various random sampling methods, such as random sampling, k-fold cross-validation, leave-one-out, and bootstrap, used to generate the validation set. This process introduced certain biases, as the dataset and validation set distributions were relatively similar, which might have limited the generalizability of the model. In addition, biases possibly arose during the random sampling process. External validation refers to situations where the dataset and validation set came from different datasets, particularly with differences in factors such as underlying conditions. External validation demonstrated the model’s generalizability and verifiability. Particularly in radiomics, external validation was worth attempting due to challenges stemming from image parameters and vector knowledge from region of heterogeneity segmentation, which could differ significantly between datasets. If a model performed exceptionally well in external validation, it indicated high robustness. In this review, it was found that most radiomics studies in ICC primarily focused on internal validation, lacking substantial external validation to support their feasibility. Consequently, this imposed certain limitations in interpreting the results. Although 12 studies based on external validation were included, it was found that radiomics in ICC involved many task types, and few external validation studies existed for each task type. Therefore, subgroup analysis of the validation set generation method was not performed, nor was there further discussion.

Moreover, the studies included manufacturers such as Siemens, Philips, and others, but some original studies did not specify the exact manufacturers in detail. In addition, some studies included images from multiple manufacturers. Due to limitations in the number of studies and varying reporting methods, an in-depth discussion of radiomics applications in ICC under different or multiple manufacturers was not conducted. Parameters may have affected the results, but the categories and quantities of parameters included in the studies varied widely, and many studies did not present imaging protocols or acquisition parameters. As a result, the discussion on imaging parameters is also limited and is shown only in the tables.

Our study shows that radiomics demonstrates ideal results in the diagnosis and prognosis of ICC and holds significant promise for future clinical practice. However, we find that integrating radiomics into PACS systems to enable an intelligent diagnostic process still presents challenges. First, the segmentation of radiomics images is a crucial process, and most current studies rely on manual segmentation. As a result, differences in segmentation exist depending on the experience and expertise of the radiologists, which may impact the model’s fitting or application performance. Therefore, prioritizing DL-based automatic segmentation is imperative. To improve interpretability, the development of more highly automated ML models or DL diagnostic systems represents a promising direction for future research. In conclusion, future research should consider the accuracy of image segmentation after integration into PACS systems, develop intelligent tools to achieve highly accurate image segmentation, and promote the intelligent reading of subsequent imaging data. Furthermore, potential regulatory issues will need to be considered, including how to avoid patient information leakage and address ethical concerns related to new systems and processes introduced during evaluation.

### Limitations

There are several limitations in our study. First, gray literature was not included due to its specific circulation channels, which may lead to publication bias. Moreover, the heterogeneity in all studies could not be fully excluded. In addition to CT types and features, as well as ML models, the heterogeneity may be attributed to variations in image segmentation, feature extraction, pathological types, and modeling algorithms. Nevertheless, because of limited studies in these subgroups, it was not possible to elucidate these detailed features. Therefore, the quantitative analysis results should be interpreted carefully. In radiomics research, the public disclosure of feature selection, dimensionality reduction techniques, and hyperparameter tuning enhanced the transparency and readability of the studies. Since many original studies did not provide detailed information on hyperparameter tuning, including manufacturer and parameter descriptions, this research listed only the specific details in tables without further discussion. This represented another limitation of existing radiomics studies [34,35]. Third, although plenty of papers were included, subgroup analyses were conducted based on different models, image sources, and tasks. Hence, a limited number of original studies were included for certain types of tasks and images. Due to relatively few studies on some models, it is difficult to discuss the performance differences among various models. Finally, few studies compare the diagnostic performance of radiomics with that of radiologists, and thereby, it is infeasible to elucidate the accuracy of radiomics versus human assessments.

### Conclusions

Our study unravels that radiomics is broadly used in ICC, particularly in diagnosing ICC, MVI, genetic mutations, PNI, LN positivity, and TLSs, and predicting recurrence and OS. It is a noninvasive technique for tumor diagnosis. However, due to the reliance on manual segmentation, equipment parameters, and expert knowledge, further studies should aim to include more cases. Moreover, research on DL in this field is still insufficient. Future studies should incorporate more cases and focus on identifying heterogeneity. It is also essential to develop intelligent image-based reading programs for ICC, which could act as an adjunct technique for the diagnosis and treatment of ICC.

## Appendix

Multimedia Appendix 1 PRISMA 2020 checklist.

Multimedia Appendix 2 Additional tables.

## Abbreviations

AUROC: area under the receiver operating characteristic curve
CEUS: contrast-enhanced ultrasonography
CF: clinical feature
CT: computed tomography
CYFRA21-1: cytokeratin-19 fragment
DL: deep learning
DOR: diagnostic odds ratio
HCC: hepatocellular carcinoma
ICC: intrahepatic cholangiocarcinoma
LN: lymph node
MeSH: Medical Subject Headings
ML: machine learning
MRI: magnetic resonance imaging
MVI: microvascular invasion
OS: overall survival
PET: positron emission tomography
PFS: progression-free survival
PNI: perineural invasion
PRISMA: Preferred Reporting Items for Systematic Reviews and Meta-Analyses
QUADAS-2: Quality Assessment of Diagnostic Accuracy Studies 2
SEN: sensitivity
SPC: specificity
TLS: tertiary lymphoid structure
US: ultrasonography

## Data Availability

The original contributions presented in the study are included in the paper, and further inquiries can be directed to the corresponding author.
