# Supplementary material for: The Application Status of Radiomics-Based Machine Learning in Intrahepatic Cholangiocarcinoma: Systematic Review and Meta-Analysis
Source: J Med Internet Res. 2025 May 5;27:e69906. doi: 10.2196/69906 (PMC12089883; doi:10.2196/69906)
Supplement: Multimedia Appendix 2 [file jmir_v27i1e69906_app2.zip › Table S4-S19.docx]

**Table S4** C-index of radiomics-based machine learning for diagnosing ICC

| **Model variables** | **Model type** | **Training set** | | | **Validation set** | | |
| --- | --- | --- | --- | --- | --- | --- | --- |
|  |  | **n** | **Number of cases** | **c-index(95%CI)** | **n** | **Number of cases** | **c-index(95%CI)** |
| Clinical features |  |  |  |  |  |  |  |
|  | LR | 7 | 795 | 0.846(0.795~0.896) | 8 | 388 | 0.800(0.756~0.844) |
|  | SVM | 2 | 242 | 0.823(0.773~0.874) | 5 | 296 | 0.701(0.645~0.757) |
|  | RF | 2 | 220 | 0.706(0.422~0.991) | 1 | 41 | 0.750(0.608~0.892) |
|  | Overall | 11 | 1257 | 0.814(0.760~0.869 ) | 14 | 725 | 0.762(0.728~0.796) |
| Radiomics |  |  |  |  |  |  |  |
| CT | DL | 3 | 1223 | 0.939(0.891~0.988) | 6 | 458 | 0.937(0.893~0.982) |
|  | RF | 1 | 346 | 0.913(0.874~0.952) | 2 | 148 | 0.834(0.754~0.914) |
|  | LR | 5 | 724 | 0.806(0.758~0.854) | 6 | 310 | 0.807(0.759~0.855) |
|  | LASSO | 1 | 346 | 0.759(0.697~0.821) | 2 | 148 | 0.755(0.660~0.850) |
|  | SVM | 3 | 590 | 0.888(0.854~0.923) | 2 | 148 | 0.614(0.504~0.725) |
|  | Overall | 13 | 3229 | 0.863(0.823~0.902 ) | 18 | 1212 | 0.836(0.787~0.885) |
| MRI |  |  |  |  |  |  |  |
|  | LR | 4 | 500 | 0.892(0.861~0.923) | 5 | 265 | 0.883(0.841~0.924) |
|  | SVM | 1 | 93 | 0.850(0.774~0.926) | 2 | 66 | 0.748(0.638~0.858) |
|  | RF | 2 | 217 | 0.900(0.857~0.944) | 2 | 94 | 0.806(0.715~0.897) |
|  | DL |  |  |  | 1 | 25 | 0.968(0.903~1.000) |
|  | Overall | 7 | 810 | 0.891(0.869 ~0.914 ) | 10 | 450 | 0.862(0.814~0.909) |
| US |  |  |  |  |  |  |  |
|  | RF | 2 | 448 | 0.927(0.847~1.000) | 2 | 116 | 0.889(0.753~1.000) |
|  | SVM | 1 | 149 | 0.860(0.801~0.919) | 2 | 77 | 0.793(0.683~0.903) |
|  | DL |  |  |  | 2 | 590 | 0.891(0.786~0.996) |
|  | Overall | 3 | 597 | 0.907(0.839~0.976) | 6 | 783 | 0.872(0.814~0.930) |
| pet-CT |  |  |  |  |  |  |  |
|  | Overall | 1 | 127 | 0.830(0.763 ~0.897) |  |  |  |
| Overall |  | 24 | 4763 | 0.876(0.851 ~0.901) | 34 | 2445 | 0.853(0.824~0.882) |
| Radiomics+clinical features |  |  |  |  |  |  |  |
| CT |  |  |  |  |  |  |  |
|  | LR | 5 | 500 | 0.913(0.886~0.940) | 5 | 213 | 0.910(0.873~0.946) |
|  | RF | 1 | 129 | 0.997(0.990~1.000) | 1 | 29 | 0.781(0.611~0.951) |
|  | Overall | 6 | 629 | 0.923(0.873~0.972) | 6 | 242 | 0.896(0.853~0.939) |
| MRI |  |  |  |  |  |  |  |
|  | SVM | 1 | 93 | 0.950(0.906~0.994) | 1 | 41 | 0.900(0.807~0.993) |
|  | LR | 6 | 626 | 0.951(0.933~0.969) | 5 | 265 | 0.930(0.900~0.960) |
|  | RF | 2 | 217 | 0.969(0.944~0.993) | 2 | 94 | 0.896(0.829~0.964) |
|  | Overall | 9 | 936 | 0.957(0.944~0.970) | 8 | 400 | 0.922(0.896~0.949) |
| US |  |  |  |  |  |  |  |
|  | RF | 1 | 224 | 0.975(0.958~0.992) | 1 | 58 | 0.971(0.939~1.000) |
|  | LR | 1 | 95 | 0.920(0.874~0.966) | 1 | 42 | 0.728(0.606~0.850) |
|  | SVM | 1 | 149 | 0.975(0.946~1.000) | 2 | 77 | 0.912(0.840~0.983) |
|  | DL |  |  |  | 1 | 50 | 0.924(0.863~0.984) |
|  | Overall | 3 | 468 | 0.964(0.937~0.990) | 5 | 227 | 0.902(0.835~0.969) |
| pet-CT |  |  |  |  |  |  |  |
|  | RF | 1 | 127 | 0.800(0.728~0.872) |  |  |  |
|  | Overall | 1 | 127 | 0.800(0.728~0.872) |  |  |  |
| Overall |  | 19 | 2225 | 0.940(0.922~0.959) | 19 | 869 | 0.912(0.889~0.935) |

**Table S5** Sensitivity and specificity of radiomics-based machine learning for diagnosing ICC

| **Model variables** | **Training set** | | | **Validation set** | | |
| --- | --- | --- | --- | --- | --- | --- |
|  | **n** | **sen(95%CI)** | **spe(95%CI)** | **n** | **sen(95%CI)** | **spe(95%CI)** |
| Clinical features | 10 | 0.79(0.72~0.84) | 0.80(0.71~0.87) | 14 | 0.72(0.66~0.77) | 0.72(0.66~0.78) |
| Radiomics |  |  |  |  |  |  |
| CT | 13 | 0.83(0.80~0.86) | 0.88(0.79~0.94) | 19 | 0.82(0.71~0.89) | 0.90(0.81~0.95) |
| MRI | 7 | 0.82(0.75~0.87) | 0.84(0.77~0.88) | 8 | 0.74(0.66~0.82) | 0.82(0.74~0.87) |
| US | 3 | 0.77-0.90 | 0.75-0.88 | 6 | 0.79(0.67~0.88) | 0.92(0.74~0.98) |
| Overall | 23 | 0.83(0.80~0.86) | 0.86(0.81~0.90) | 33 | 0.80(0.73~0.85) | 0.88(0.83~0.92) |
| Radiomics+clinical features |  |  |  |  |  |  |
| CT | 5 | 0.88(0.75~0.95) | 0.89(0.84~0.93) | 6 | 0.73(0.65~0.80) | 0.88(0.81~0.93) |
| MRI | 9 | 0.90(0.85~0.94) | 0.91(0.86~0.95) | 8 | 0.77(0.70~0.83) | 0.91(0.86~0.95) |
| US | 3 | 0.84-0.97 | 0.85-0.92 | 5 | 0.83(0.71~0.91) | 0.88(0.80~0.93) |
| Overall | 17 | 0.90(0.85~0.93) | 0.90(0.87~0.93) | 19 | 0.77(0.72~0.81) | 0.90(0.86~0.92) |

**Table S6** C-index of radiomics-based machine learning for diagnosing MVI

| **Model variables** | **Model type** | **Training set** | | | **Validation set** | | |
| --- | --- | --- | --- | --- | --- | --- | --- |
|  |  | **n** | **Number of cases** | **c-index(95%CI)** | **n** | **Number of cases** | **c-index(95%CI)** |
| Clinical features |  |  |  |  |  |  |  |
|  | LR | 4 | 615 | 0.715(0.659~0.772) | 2 | 101 | 0.645(0.440~0.850) |
|  | SVM | 1 | 130 | 0.726(0.635~0.817) | 1 | 33 | 0.483(0.277~0.689) |
|  | RF | 2 | 181 | 0.717(0.645~0.788) | 1 | 33 | 0.578(0.374~0.782) |
|  | Overall | 7 | 926 | 0.717(0.679~0.755) | 4 | 167 | 0.599(0.471~0.727) |
| Radiomics |  |  |  |  |  |  |  |
| MRI |  |  |  |  |  |  |  |
|  | MPFCNN |  |  |  | 2 | 158 | 0.869(0.799~0.939) |
|  | SVM | 1 | 130 | 0.947(0.905~0.989) | 2 | 57 | 0.848(0.739~0.957) |
|  | LR | 2 | 297 | 0.881(0.740~1.000) | 3 | 125 | 0.825(0.748~0.902) |
|  | RF | 1 | 130 | 0.967(0.934~1.000) | 2 | 57 | 0.859(0.748~0.971) |
|  | Overall | 4 | 557 | 0.925(0.872~0.977) | 9 | 397 | 0.851(0.808~0.894) |
| pet-CT |  |  |  |  |  |  |  |
|  | RF | 1 | 51 | 0.880(0.802~0.958) |  |  |  |
|  | Overall | 1 | 51 | 0.880(0.802~0.958) |  |  |  |
| Overall |  | 5 | 608 | 0.918(0.870~0.966) | 9 | 397 | 0.851(0.808~0.894) |
| Radiomics+clinical features |  |  |  |  |  |  |  |
| CT |  |  |  |  |  |  |  |
|  | LR | 3 | 732 | 0.802(0.660~0.945) |  |  |  |
|  | Overall | 3 | 732 | 0.802(0.660~0.945) |  |  |  |
| MRI |  |  |  |  |  |  |  |
|  | RF | 1 | 130 | 0.988(0.968~1.000) | 2 | 57 | 0.851(0.742~0.959) |
|  | LR | 2 | 297 | 0.893(0.769~1.000) | 3 | 125 | 0.848(0.778~0.917) |
|  | LASSO | 1 | 88 | 0.873(0.796~0.950) | 1 | 38 | 0.850(0.709~0.991) |
|  | SVM | 1 | 130 | 0.898(0.840~0.956) | 2 | 57 | 0.851(0.742~0.960) |
|  | Overall | 5 | 645 | 0.913(0.855~0.972) | 8 | 277 | 0.849(0.801~0.897) |
| US |  |  |  |  |  |  |  |
|  | SVM | 1 | 61 | 0.699(0.567~0.831) | 1 | 61 | 0.756(0.634~0.878) |
|  | Overall | 1 | 61 | 0.699(0.567~0.831) | 1 | 61 | 0.756(0.634~0.878) |
| pet-CT |  |  |  |  |  |  |  |
|  | LR | 2 | 148 | 0.876(0.825~0.927) |  |  |  |
|  | RF | 1 | 51 | 0.900(0.828~0.972) |  |  |  |
|  | Overall | 3 | 199 | 0.884(0.843~0.926) |  |  |  |
| Overall |  | 12 | 1637 | 0.881(0.834~0.928) | 9 | 338 | 0.836(0.792~0.881) |

**Table S7** Sensitivity and specificity of radiomics-based machine learning for diagnosing MVI

| **Model variables** | **Training set** | | | **Validation set** | | |
| --- | --- | --- | --- | --- | --- | --- |
|  | **n** | **sen(95%CI)** | **spe(95%CI)** | **n** | **sen(95%CI)** | **spe(95%CI)** |
| Clinical features | 5 | 0.55(0.38~0.72) | 0.79(0.66~0.88) | 3 | 0.1-0.58 | 0.61-0.96 |
| Radiomics |  |  |  |  |  |  |
| MRI | 4 | 0.83(0.68~0.91) | 0.90(0.64~0.98) | 11 | 0.75(0.68~0.81) | 0.84(0.78~0.89) |
| Overall | 4 | 0.83(0.68~0.91) | 0.90(0.64~0.98) | 11 | 0.75(0.68~0.81) | 0.84(0.78~0.89) |
| Radiomics+clinical features |  |  |  |  |  |  |
| CT | 3 | 0.77-0.78 | 0.66-0.71 |  |  |  |
| MRI | 5 | 0.86(0.77~0.92) | 0.88(0.76~0.95) | 8 | 0.77(0.67~0.84) | 0.88(0.79~0.94) |
| US | 1 | 0.55 | 0.93 |  |  |  |
| pet-CT | 3 | 0.75-0.82 | 0.79-0.8 |  |  |  |
| Overall | 12 | 0.80(0.75~0.84) | 0.83(0.75~0.89) | 8 | 0.77(0.67~0.84) | 0.88(0.79~0.94) |

**Table S8** C-index of radiomics-based machine learning for diagnosing gene mutations

| **Model variables** | **Model type** | **Training set** | | | **Validation set** | | |
| --- | --- | --- | --- | --- | --- | --- | --- |
|  |  | **n** | **Number of cases** | **c-index(95%CI)** | **n** | **Number of cases** | **c-index(95%CI)** |
| Clinical features |  |  |  |  |  |  |  |
|  | LR | 4 | 259 | 0.677(0.543~0.812) | 2 | 103 | 0.587(0.491~0.683) |
|  | RF |  |  |  | 2 | 103 | 0.587(0.491~0.683) |
|  | Overall | 4 | 259 | 0.677(0.543~0.812) | 4 | 206 | 0.594(0.541~0.646) |
| Radiomics |  |  |  |  |  |  |  |
| CT |  |  |  |  |  |  |  |
|  | LR | 3 | 206 | 0.902(0.847~0.956) |  |  |  |
|  | SVM | 10 | 1380 | 0.774(0.686~0.861) |  |  |  |
|  | Overall | 13 | 1586 | 0.801(0.731~0.870) |  |  |  |
| MRI |  |  |  |  |  |  |  |
|  | LR | 1 | 53 | 0.770(0.570~0.970) |  |  |  |
|  | RF | 1 | 124 | 0.813(0.694~0.933) | 2 | 103 | 0.902(0.855~0.949) |
|  | Overall | 2 | 177 | 0.802(0.699~0.904) | 2 | 103 | 0.902(0.855~0.949) |
| Overall |  | 15 | 1763 | 0.800(0.737~0.864) |  |  |  |
| Radiomics+clinical features |  |  |  |  |  |  |  |
| CT |  |  |  |  |  |  |  |
|  | LR | 3 | 206 | 0.961(0.926~0.995) |  |  |  |
|  | Overall | 3 | 206 | 0.961(0.926~0.995) |  |  |  |
| MRI |  |  |  |  |  |  |  |
|  | LR | 1 | 53 | 0.909(0.803~1.000) | 2 | 74 | 0.887(0.816~0.958) |
|  | RF | 1 | 124 | 0.807(0.680~0.934) | 1 | 54 | 0.920(0.871~0.969) |
|  | Overall | 2 | 177 | 0.864(0.765~0.963) | 3 | 128 | 0.909(0.869~0.950) |
| US |  |  |  |  |  |  |  |
|  | LR |  |  |  | 1 | 90 | 0.848(0.788~0.908) |
|  | Overall | 1 | 90 | 0.804(0.737~0.871) | 1 | 90 | 0.848(0.788~0.908) |
| Overall |  | 6 | 473 | 0.892(0.822~0.962) | 4 | 218 | 0.889(0.851~0.927) |

**Table S9** Sensitivity and specificity of radiomics-based machine learning for diagnosing gene mutations

| **Model variables** | **Training set** | | | **Validation set** | | |
| --- | --- | --- | --- | --- | --- | --- |
|  | **n** | **sen(95%CI)** | **spe(95%CI)** | **n** | **sen(95%CI)** | **spe(95%CI)** |
| Clinical features | 2 | 0.20-0.73 | 0.60-0.93 | 4 | 0.98 (0.82~1.00) | 0.02 (0.00~0.31) |
| Radiomics |  |  |  |  |  |  |
| CT | 13 | 0.84 (0.66~0.94) | 0.92 (0.87~0.95) |  |  |  |
| MRI | 2 | 0.75-0.96 | 0.67-0.89 | 2 | 0.909-0.911 | 0.69-0.81 |
| Overall | 15 | 0.85 (0.69~0.93) | 0.91 (0.86~0.94) | 2 | 0.909-0.911 | 0.69-0.81 |
| Radiomics+clinical features |  |  |  |  |  |  |
| CT | 3 | 0.56-0.8 | 0.96-0.98 |  |  |  |
| MRI | 2 | 0.74-1 | 0-0.77 | 2 | 0.82-0.86 | 0.33-0.76 |
| US | 1 | 1 | 0.2 | 1 | 0.96 | 0.5 |
| Overall | 6 | 0.93 (0.55~0.99) | 0.69 (0.11~0.98) | 3 | 0.82-0.96 | 0.33-0.76 |

**Table S10** C-index of radiomics-based machine learning for diagnosing PNI

| **Model variables** | **Model type** | **Training set** | | | **Validation set** | | |
| --- | --- | --- | --- | --- | --- | --- | --- |
|  |  | **n** | **Number of cases** | **c-index(95%CI)** | **n** | **Number of cases** | **c-index(95%CI)** |
| Clinical features |  |  |  |  |  |  |  |
|  | LR | 2 | 272 | 0.731(0.598~0.864) | 3 | 133 | 0.746(0.604~0.889) |
|  | Overall | 2 | 272 | 0.731(0.598~0.864) | 3 | 133 | 0.746(0.604~0.889) |
| Radiomics+clinical features |  |  |  |  |  |  |  |
| CT |  |  |  |  |  |  |  |
|  | LR | 1 | 136 | 0.855(0.794~0.916) | 2 | 107 | 0.841(0.761~0.921) |
|  | Overall | 1 | 136 | 0.855(0.794~0.916) | 2 | 107 | 0.841(0.761~0.921) |
| US |  |  |  |  |  |  |  |
|  | SVM | 1 | 56 | 0.930(0.858~1.000) | 1 | 56 | 0.894(0.806~0.982) |
|  | Overall | 1 | 56 | 0.930(0.858~1.000) | 1 | 56 | 0.894(0.806~0.982) |
| Overall |  | 2 | 192 | 0.890(0.817~0.964) | 3 | 163 | 0.865(0.806~0.924) |

**Table S11** Sensitivity and specificity of radiomics-based machine learning for diagnosing PNI

| **Model variables** | **Training set** | | | **Validation set** | | |
| --- | --- | --- | --- | --- | --- | --- |
|  | **n** | **sen(95%CI)** | **spe(95%CI)** | **n** | **sen(95%CI)** | **spe(95%CI)** |
| Clinical features | 2 | 0.61-0.73 | 0.74-0.79 | 3 | 0.43-1 | 0.32-1 |
| Radiomics+clinical features |  |  |  |  |  |  |
| CT | 1 | 0.85 | 0.73 | 2 | 0.67-0.86 | 0.79-0.95 |
| US | 1 | 0.85 | 0.98 |  |  |  |
| Overall | 2 | 0.84-0.85 | 0.73-0.98 | 2 | 0.67-0.86 | 0.79-0.95 |

**Table S12** C-index of radiomics-based machine learning for diagnosing positive LN

| **Model variables** | **Model type** | **Training set** | | | **Validation set** | | |
| --- | --- | --- | --- | --- | --- | --- | --- |
|  |  | **n** | **Number of cases** | **c-index(95%CI)** | **n** | **Number of cases** | **c-index(95%CI)** |
| Clinical features |  |  |  |  |  |  |  |
|  | LR | 1 | 170 | 0.870(0.825~0.915) | 2 | 126 | 0.798(0.721~0.875) |
|  | SVM | 1 | 106 | 0.658(0.564~0.752) | 1 | 42 | 0.673(0.524~0.822) |
|  | Overall | 2 | 276 | 0.768(0.561~0.976) | 3 | 168 | 0.768(0.682~0.853) |
| Radiomics |  |  |  |  |  |  |  |
| CT |  |  |  |  |  |  |  |
|  | LR |  |  |  | 10 | 630 | 0.768(0.721~0.814) |
|  | RF | 5 | 850 | 0.822(0.727~0.916) |  |  |  |
|  | Overall | 5 | 850 | 0.822(0.727~0.916) | 10 | 630 | 0.768(0.721~0.814) |
| MRI |  |  |  |  |  |  |  |
|  | SVM | 1 | 106 | 0.788(0.706~0.870) | 1 | 42 | 0.787(0.655~0.919) |
|  | Overall | 1 | 106 | 0.788(0.706~0.870) | 1 | 42 | 0.787(0.655~0.919) |
| Overall |  | 6 | 956 | 0.817(0.732~0.901) | 11 | 672 | 0.769(0.726~0.813) |
| Radiomics+clinical features |  |  |  |  |  |  |  |
| CT |  |  |  |  |  |  |  |
|  | LR | 1 | 170 | 0.980(0.965~0.995) | 2 | 126 | 0.918(0.876~0.959) |
|  | Overall | 1 | 170 | 0.980(0.965~0.995) | 2 | 126 | 0.918(0.876~0.959) |
| MRI |  |  |  |  |  |  |  |
|  | SVM | 1 | 106 | 0.842(0.768~0.916) | 1 | 42 | 0.870(0.758~0.982) |
|  | Overall | 1 | 106 | 0.842(0.768~0.916) | 1 | 42 | 0.870(0.758~0.982) |
| Overall |  | 2 | 276 | 0.916(0.781~1.000) | 3 | 168 | 0.912(0.873~0.951) |

**Table S13** Sensitivity and specificity of radiomics-based machine learning for diagnosing positive LN

| **Model variables** | **Training set** | | | **Validation set** | | |
| --- | --- | --- | --- | --- | --- | --- |
|  | **n** | **sen(95%CI)** | **spe(95%CI)** | **n** | **sen(95%CI)** | **spe(95%CI)** |
| Clinical features | 2 | 0.64-0.91 | 0.67-0.68 | 3 | 0.71-0.89 | 0.41-0.74 |
| Radiomics |  |  |  |  |  |  |
| CT | 5 | 0.68(0.53~0.80) | 0.87(0.79~0.92) | 10 | 0.68(0.52~0.81) | 0.77(0.64~0.86) |
| MRI | 1 | 0.66 | 0.8 | 1 | 0.53 | 0.91 |
| Overall | 6 | 0.68(0.55~0.78) | 0.86(0.79~0.91) | 11 | 0.67(0.52~0.79) | 0.79(0.67~0.87) |
| Radiomics+clinical features |  |  |  |  |  |  |
| CT | 1 | 0.87 | 0.97 | 2 | 0.79-0.88 | 0.82-0.9 |
| MRI | 1 | 0.89 | 0.58 | 1 | 0.89 | 0.70 |
| Overall | 2 | 0.87-0.89 | 0.58-0.97 | 3 | 0.79-0.89 | 0.70-0.90 |

**Table S14** C-index of radiomics-based machine learning for diagnosing TLSs

| **Model variables** | **Model type** | **Training set** | | | **Validation set** | | |
| --- | --- | --- | --- | --- | --- | --- | --- |
|  |  | **n** | **Number of cases** | **c-index(95%CI)** | **n** | **Number of cases** | **c-index(95%CI)** |
| Clinical features |  |  |  |  |  |  |  |
|  | LR | 1 | 86 | 0.750(0.630~0.870) | 1 | 30 | 0.710(0.485~0.935) |
|  | Overall | 1 | 86 | 0.750(0.630~0.870) | 1 | 30 | 0.710(0.485~0.935) |
| Radiomics |  |  |  |  |  |  |  |
| CT |  |  |  |  |  |  |  |
|  | LR | 1 | 86 | 0.820(0.730~0.910) | 1 | 30 | 0.860(0.735~0.985) |
|  | Overall | 1 | 86 | 0.820(0.730~0.910) | 1 | 30 | 0.860(0.735~0.985) |
| MRI |  |  |  |  |  |  |  |
|  | LR | 2 | 210 | 0.721(0.466~0.976) | 4 | 174 | 0.704(0.541~0.867) |
|  | Overall | 2 | 210 | 0.721(0.466~0.976) | 4 | 174 | 0.704(0.541~0.867) |
| Overall |  | 3 | 296 | 0.754(0.592~0.915) | 5 | 204 | 0.735(0.589~0.881) |
| Radiomics+clinical features |  |  |  |  |  |  |  |
| CT |  |  |  |  |  |  |  |
|  | LR | 1 | 86 | 0.850(0.775~0.925) | 1 | 30 | 0.880(0.760~1.000) |
|  | Overall | 1 | 86 | 0.850(0.775~0.925) | 1 | 30 | 0.880(0.760~1.000) |
| Overall |  | 1 | 86 | 0.850(0.775~0.925) | 1 | 30 | 0.880(0.760~1.000) |

**Table S15** Sensitivity and specificity of radiomics-based machine learning for diagnosing TLSs

| **Model variables** | **Training set** | | | **Validation set** | | |
| --- | --- | --- | --- | --- | --- | --- |
|  | **n** | **sen(95%CI)** | **spe(95%CI)** | **n** | **sen(95%CI)** | **spe(95%CI)** |
| Clinical features | 1 | 0.76 | 0.8 | 1 | 0.8 | 0.76 |
| Radiomics |  |  |  |  |  |  |
| CT | 1 | 0.89 | 0.69 | 1 | 1 | 0.7 |
| MRI | 2 | 0.29-0.68 | 0.88-0.91 | 4 | 0.43(0.21~0.68) | 0.93(0.86~0.96) |
| Overall | 3 | 0.29-0.89 | 0.69-0.91 | 5 | 0.61(0.22~0.89) | 0.90(0.80~0.95) |
| Radiomics+clinical features |  |  |  |  |  |  |
| CT | 1 | 0.57 | 0.93 | 1 | 0.6 | 0.93 |
| Overall | 1 | 0.57 | 0.93 | 1 | 0.6 | 0.93 |

**Table S16** C-index of radiomics-based machine learning for predicting OS

| **Model variables** | **Model type** | **Training set** | | | **Validation set** | | |
| --- | --- | --- | --- | --- | --- | --- | --- |
|  |  | **n** | **Number of cases** | **c-index(95%CI)** | **n** | **Number of cases** | **c-index(95%CI)** |
| Clinical features |  |  |  |  |  |  |  |
|  | COX | 4 | 631 | 0.726(0.662~0.790) | 1 | 43 | 0.670(0.565~0.775) |
|  | Overall | 4 | 631 | 0.726(0.662~0.790) | 1 | 43 | 0.670(0.565~0.775) |
| Radiomics+clinical features |  |  |  |  |  |  |  |
| CT |  |  |  |  |  |  |  |
|  | COX | 7 | 1367 | 0.766(0.744~0.789) | 1 | 24 | 0.684(0.527~0.841) |
|  | Overall | 7 | 1367 | 0.766(0.744~0.789) | 1 | 24 | 0.684(0.527~0.841) |
| US |  |  |  |  |  |  |  |
|  | COX | 1 | 127 | 0.740(0.695~0.785) | 1 | 43 | 0.800(0.725~0.875) |
|  | Overall | 1 | 127 | 0.740(0.695~0.785) | 1 | 43 | 0.800(0.725~0.875) |
| pet-CT |  |  |  |  |  |  |  |
|  | COX | 2 | 148 | 0.810(0.745~0.876) |  |  |  |
|  | Overall | 2 | 148 | 0.810(0.745~0.876) |  |  |  |
| Overall |  | 10 | 1642 | 0.765(0.746~0.785) | 2 | 67 | 0.763(0.657~0.869) |

**Table S17** Sensitivity and specificity of radiomics-based machine learning for predicting OS

| **Model variables** | **Training set** | | | **Validation set** | | |
| --- | --- | --- | --- | --- | --- | --- |
|  | **n** | **sen(95%CI)** | **spe(95%CI)** | **n** | **sen(95%CI)** | **spe(95%CI)** |
| Radiomics+clinical features |  |  |  |  |  |  |
| CT | 1 | 0.64 | 0.69 | 1 | 0.69 | 0.61 |
| Overall |  |  |  |  |  |  |

**Table S18** C-index of radiomics-based machine learning for predicting recurrence

| **Model variables** | **Model type** | **Training set** | | | **Validation set** | | |
| --- | --- | --- | --- | --- | --- | --- | --- |
|  |  | **n** | **Number of cases** | **c-index(95%CI)** | **n** | **Number of cases** | **c-index(95%CI)** |
| Clinical features |  |  |  |  |  |  |  |
|  | MRMR-GBM |  |  |  | 1 | 53 | 0.565(0.407~0.723) |
|  | COX | 5 | 733 | 0.793(0.744~0.842) | 5 | 241 | 0.799(0.745~0.852) |
|  | LR | 2 | 137 | 0.690(0.614~0.767) |  |  |  |
|  | LightGBM | 2 | 280 | 0.727(0.672~0.781) | 6 | 342 | 0.687(0.636~0.737) |
|  | Overall | 9 | 1150 | 0.761(0.720~0.802) | 12 | 636 | 0.727(0.681~0.773) |
| Radiomics |  |  |  |  |  |  |  |
| CT |  |  |  |  |  |  |  |
|  | LR | 1 | 90 | 0.720(0.631~0.809) | 1 | 37 | 0.860(0.760~0.960) |
|  | SVM | 1 | 90 | 0.740(0.654~0.826) | 1 | 37 | 0.880(0.787~0.973) |
|  | COX | 1 | 97 | 0.870(0.800~0.940) | 1 | 41 | 0.810(0.690~0.930) |
|  | AdaBoost | 2 | 278 | 0.755(0.745~0.765) |  |  |  |
|  | RF | 1 | 90 | 0.700(0.609~0.791) | 1 | 37 | 0.870(0.773~0.967) |
|  | Bayes | 1 | 90 | 0.780(0.699~0.861) | 1 | 37 | 0.850(0.746~0.954) |
|  | LightGBM | 2 | 230 | 0.767(0.544~0.989) | 4 | 208 | 0.774(0.712~0.836) |
|  | XGBoost | 1 | 90 | 0.710(0.620~0.800) | 1 | 37 | 0.820(0.707~0.933) |
|  | MRMR-GBM | 1 | 124 | 0.802(0.728~0.877) | 1 | 53 | 0.781(0.655~0.907) |
|  | NN | 1 | 90 | 0.740(0.654~0.826) | 1 | 37 | 0.900(0.815~0.985) |
|  | Overall | 12 | 1269 | 0.759(0.748~0.769) | 12 | 524 | 0.835(0.803~0.866) |
| MRI |  |  |  |  |  |  |  |
|  | LR | 8 | 468 | 0.827(0.790~0.864) | 2 | 279 | 0.756(0.700~0.811) |
|  | Overall | 8 | 468 | 0.827(0.790~0.864) | 2 | 279 | 0.756(0.700~0.811) |
| MRI+CT |  |  |  |  |  |  |  |
|  | COX | 3 | 477 | 0.792(0.754~0.830) | 3 | 150 | 0.816(0.749~0.882) |
|  | Overall | 3 | 477 | 0.792(0.754~0.830) | 3 | 150 | 0.816(0.749~0.882) |
| Overall |  | 23 | 2214 | 0.766(0.756~0.776) | 17 | 953 | 0.816(0.790~0.841) |
| Radiomics+clinical features |  |  |  |  |  |  |  |
| CT |  |  |  |  |  |  |  |
|  | Resnet50 |  |  |  | 1 | 41 | 0.998(0.986~1.000) |
|  | XGBoost |  |  |  | 2 | 74 | 0.780(0.694~0.867) |
|  | SVM | 1 | 90 | 0.780(0.699~0.861) | 1 | 37 | 0.860(0.760~0.960) |
|  | LightGBM | 2 | 230 | 0.871(0.661~1.000) | 4 | 208 | 0.864(0.815~0.913) |
|  | Bayes | 1 | 90 | 0.840(0.770~0.910) | 1 | 37 | 0.900(0.815~0.985) |
|  | NN | 1 | 90 | 0.830(0.758~0.902) | 1 | 37 | 0.840(0.733~0.947) |
|  | AdaBoost | 1 | 139 | 0.780(0.778~0.782) |  |  |  |
|  | RF | 1 | 90 | 0.770(0.688~0.852) | 1 | 37 | 0.880(0.787~0.973) |
|  | COX | 1 | 97 | 0.880(0.820~0.940) | 1 | 41 | 0.840(0.730~0.950) |
|  | LR | 1 | 90 | 0.800(0.723~0.877) | 1 | 37 | 0.870(0.773~0.967) |
|  | Overall | 9 | 916 | 0.826(0.757~0.895) | 13 | 549 | 0.866(0.809~0.923) |
| MRI |  |  |  |  |  |  |  |
|  | LR | 2 | 186 | 0.894(0.845~0.943) | 2 | 279 | 0.846(0.800~0.892) |
|  | Overall | 2 | 186 | 0.894(0.845~0.943) | 2 | 279 | 0.846(0.800~0.892) |
| Overall |  | 11 | 1102 | 0.837(0.775~0.898) | 15 | 828 | 0.863(0.810~0.917) |

**Table S19** Sensitivity and specificity of radiomics-based machine learning for predicting recurrence

| **Model variables** | **Training set** | | | **Validation set** | | |
| --- | --- | --- | --- | --- | --- | --- |
|  | **n** | **sen(95%CI)** | **spe(95%CI)** | **n** | **sen(95%CI)** | **spe(95%CI)** |
| Clinical features | 5 | 0.72 (0.64~ 0.79) | 0.65 (0.57~ 0.72) | 7 | 0.64 (0.56~ 0.71) | 0.64 (0.56~ 0.71) |
| Radiomics |  |  |  |  |  |  |
| CT | 12 | 0.79 (0.75~ 0.83) | 0.87 (0.80~ 0.91) |  |  |  |
| MRI | 8 | 0.76 (0.71~ 0.81) | 0.82 (0.74~ 0.88) | 12 | 0.83 (0.77~ 0.88) | 0.80 (0.74~ 0.85) |
| US |  |  |  | 2 | 0.78-0.94 | 0.5-0.63 |
| Overall | 20 | 0.78 (0.75~ 0.82) | 0.86 (0.81~ 0.90) | 14 | 0.84 (0.78~ 0.88) | 0.76 (0.69~ 0.82) |
| Radiomics+clinical features |  |  |  |  |  |  |
| CT | 9 | 0.85 (0.77~ 0.90) | 0.75 (0.67~ 0.82) | 13 | 0.84 (0.78~ 0.89) | 0.75 (0.68~ 0.80) |
| MRI | 2 | 0.74-0.94 | 0.84-0.89 | 2 | 0.81-0.89 | 0.64-0.82 |
| Overall | 11 | 0.85 (0.78~ 0.90) | 0.77 (0.70~ 0.84) | 15 | 0.85 (0.80~ 0.88) | 0.74 (0.67~ 0.79) |
