# Supplementary material for: The Application Status of Radiomics-Based Machine Learning in Intrahepatic Cholangiocarcinoma: Systematic Review and Meta-Analysis
Source: J Med Internet Res. 2025 May 5;27:e69906. doi: 10.2196/69906 (PMC12089883; doi:10.2196/69906)
Supplement: Multimedia Appendix 2 [file jmir_v27i1e69906_app2.zip › Table S1.docx]

# Table S1 Literature search strategy

**1.Pubmed**

| Search number | Query | Results |
| --- | --- | --- |
| #1 | "Cholangiocarcinoma"[MeSH Terms] | 13,260 |
| #2 | "Cholangiocarcinomas"[Title/Abstract] OR "cholangiocellular carcinoma"[Title/Abstract] OR "cholangiocellular carcinomas"[Title/Abstract] OR "extrahepatic cholangiocarcinoma"[Title/Abstract] OR "extrahepatic cholangiocarcinomas"[Title/Abstract] OR "intrahepatic cholangiocarcinomas"[Title/Abstract] | 3,354 |
| #3 | "Cholangiocarcinoma"[MeSH Terms] OR "Cholangiocarcinomas"[Title/Abstract] OR "cholangiocellular carcinoma"[Title/Abstract] OR "cholangiocellular carcinomas"[Title/Abstract] OR "extrahepatic cholangiocarcinoma"[Title/Abstract] OR "extrahepatic cholangiocarcinomas"[Title/Abstract] OR "intrahepatic cholangiocarcinomas"[Title/Abstract] | 14,756 |
| #4 | "Radiomics"[MeSH Terms] | 1,015 |
| #5 | "Radiomics"[Title/Abstract] OR "Radiomic"[Title/Abstract] OR "radiogenomic"[Title/Abstract] OR "radiomics-based"[Title/Abstract] OR "Texture"[Title/Abstract] OR "machine learning"[Title/Abstract] OR "transfer learning"[Title/Abstract] OR "deep learning"[Title/Abstract] OR "ensemble learning"[Title/Abstract] OR "artificial intelligence"[Title/Abstract] OR "random forest"[Title/Abstract] OR "neural network"[Title/Abstract] OR "neural networks"[Title/Abstract] OR "k nearest neighbor"[Title/Abstract] OR "CNN"[Title/Abstract] OR "AlexNet"[Title/Abstract] OR "VGGNet"[Title/Abstract] OR "ResNet"[Title/Abstract] OR "GoogLeNet"[Title/Abstract] OR "support vector machine"[Title/Abstract] OR "SVM"[Title/Abstract] OR "gradient boosting machine"[Title/Abstract] OR "Nomogram"[Title/Abstract] OR "XGBoost"[Title/Abstract] OR "Adaboost"[Title/Abstract] OR "LightGBM"[Title/Abstract] OR "CatBoost"[Title/Abstract] OR "decision tree"[Title/Abstract] OR "naive bayesian"[Title/Abstract] OR "multilayer perceptron"[Title/Abstract] OR "bayesian network"[Title/Abstract] OR "prediction model"[Title/Abstract] OR "risk model"[Title/Abstract] | 413,343 |
| #6 | "Radiomics"[MeSH Terms] OR "Radiomics"[Title/Abstract] OR "Radiomic"[Title/Abstract] OR "radiogenomic"[Title/Abstract] OR "radiomics-based"[Title/Abstract] OR "Texture"[Title/Abstract] OR "machine learning"[Title/Abstract] OR "transfer learning"[Title/Abstract] OR "deep learning"[Title/Abstract] OR "ensemble learning"[Title/Abstract] OR "artificial intelligence"[Title/Abstract] OR "random forest"[Title/Abstract] OR "neural network"[Title/Abstract] OR "neural networks"[Title/Abstract] OR "k nearest neighbor"[Title/Abstract] OR "CNN"[Title/Abstract] OR "AlexNet"[Title/Abstract] OR "VGGNet"[Title/Abstract] OR "ResNet"[Title/Abstract] OR "GoogLeNet"[Title/Abstract] OR "support vector machine"[Title/Abstract] OR "SVM"[Title/Abstract] OR "gradient boosting machine"[Title/Abstract] OR "Nomogram"[Title/Abstract] OR "XGBoost"[Title/Abstract] OR "Adaboost"[Title/Abstract] OR "LightGBM"[Title/Abstract] OR "CatBoost"[Title/Abstract] OR "decision tree"[Title/Abstract] OR "naive bayesian"[Title/Abstract] OR "multilayer perceptron"[Title/Abstract] OR "bayesian network"[Title/Abstract] OR "prediction model"[Title/Abstract] OR "risk model"[Title/Abstract] | 413,344 |
| #7 | ("Cholangiocarcinoma"[MeSH Terms] OR ("Cholangiocarcinomas"[Title/Abstract] OR "cholangiocellular carcinoma"[Title/Abstract] OR "cholangiocellular carcinomas"[Title/Abstract] OR "extrahepatic cholangiocarcinoma"[Title/Abstract] OR "extrahepatic cholangiocarcinomas"[Title/Abstract] OR "intrahepatic cholangiocarcinomas"[Title/Abstract])) AND ("Radiomics"[MeSH Terms] OR ("Radiomics"[Title/Abstract] OR "Radiomic"[Title/Abstract] OR "radiogenomic"[Title/Abstract] OR "radiomics-based"[Title/Abstract] OR "Texture"[Title/Abstract] OR "machine learning"[Title/Abstract] OR "transfer learning"[Title/Abstract] OR "deep learning"[Title/Abstract] OR "ensemble learning"[Title/Abstract] OR "artificial intelligence"[Title/Abstract] OR "random forest"[Title/Abstract] OR "neural network"[Title/Abstract] OR "neural networks"[Title/Abstract] OR "k nearest neighbor"[Title/Abstract] OR "CNN"[Title/Abstract] OR "AlexNet"[Title/Abstract] OR "VGGNet"[Title/Abstract] OR "ResNet"[Title/Abstract] OR "GoogLeNet"[Title/Abstract] OR "support vector machine"[Title/Abstract] OR "SVM"[Title/Abstract] OR "gradient boosting machine"[Title/Abstract] OR "Nomogram"[Title/Abstract] OR "XGBoost"[Title/Abstract] OR "Adaboost"[Title/Abstract] OR "LightGBM"[Title/Abstract] OR "CatBoost"[Title/Abstract] OR "decision tree"[Title/Abstract] OR "naive bayesian"[Title/Abstract] OR "multilayer perceptron"[Title/Abstract] OR "bayesian network"[Title/Abstract] OR "prediction model"[Title/Abstract] OR "risk model"[Title/Abstract])) | 323 |

**2.Cochrane**

| Search number | Query | Results |
| --- | --- | --- |
| #1 | MeSH descriptor: [Cholangiocarcinoma] explode all trees | 365 |
| #2 | (Cholangiocarcinoma or Cholangiocarcinomas or Cholangiocellular Carcinoma or Cholangiocellular Carcinomas or Extrahepatic Cholangiocarcinoma or Extrahepatic Cholangiocarcinomas or Intrahepatic Cholangiocarcinomas):ti,ab,kw | 1040 |
| #3 | #1 or #2 | 1043 |
| #4 | MeSH descriptor: [Radiomics] explode all trees | 47 |
| #5 | (Radiomics or Radiomic or radiogenomic or radiomics-based or Texture or machine learning or Transfer Learning or Deep learning or Ensemble Learning or artificial intelligence or random forest or neural network or neural networks or K-Nearest Neighbor or CNN or AlexNet or VGGNet or ResNet or GoogLeNet or Support vector machine or SVM or Gradient Boosting Machine or Nomogram or XGBoost or Adaboost or LightGBM or CatBoost or Decision tree or Naive Bayesian or Multilayer perceptron or Bayesian network or Prediction model or Risk model):ti,ab,kw | 49145 |
| #6 | #4 or #5 | 49145 |
| #7 | #3 and #6 | 53 |

**3.Embase**

| Search number | Query | Results |
| --- | --- | --- |
| #1 | 'intrahepatic cholangiocarcinoma'/exp | 2106 |
| #2 | 'intrahepatic bile duct carcinoma':ab,ti OR 'intrahepatic biliary duct carcinoma':ab,ti OR 'intrahepatic cholangiocellular carcinoma':ab,ti OR 'intrahepatic cholangiocarcinoma':ab,ti | 7915 |
| #3 | #1 OR #2 | 8406 |
| #4 | 'radiomics'/exp | 11918 |
| #5 | radiomics:ab,ti OR radiomic:ab,ti OR radiogenomic:ab,ti OR 'radiomics based':ab,ti OR texture:ab,ti OR 'machine learning':ab,ti OR 'transfer learning':ab,ti OR 'deep learning':ab,ti OR 'ensemble learning':ab,ti OR 'artificial intelligence':ab,ti OR 'random forest':ab,ti OR 'neural network':ab,ti OR 'neural networks':ab,ti OR 'k-nearest neighbor':ab,ti OR cnn:ab,ti OR alexnet:ab,ti OR vggnet:ab,ti OR resnet:ab,ti OR googlenet:ab,ti OR 'support vector machine':ab,ti OR svm:ab,ti OR 'gradient boosting machine':ab,ti OR nomogram:ab,ti OR xgboost:ab,ti OR adaboost:ab,ti OR lightgbm:ab,ti OR catboost:ab,ti OR 'decision tree':ab,ti OR 'naive bayesian':ab,ti OR 'multilayer perceptron':ab,ti OR 'bayesian network':ab,ti OR 'prediction model':ab,ti OR 'risk model':ab,ti | 469033 |
| #6 | #4 OR #5 | 469752 |
| #7 | #3 AND #6 | 376 |

**4.Web of science**

| Search number | Query | Results |
| --- | --- | --- |
| #1 | Cholangiocarcinoma (Topic) or Cholangiocarcinomas (Topic) or Cholangiocellular Carcinoma (Topic) or Cholangiocellular Carcinomas (Topic) or Extrahepatic Cholangiocarcinoma (Topic) or Extrahepatic Cholangiocarcinomas (Topic) or Intrahepatic Cholangiocarcinomas (Topic) or intrahepatic cholangiocarcinoma (Topic) or intrahepatic bile duct carcinoma (Topic) or intrahepatic biliary duct carcinoma (Topic) or intrahepatic cholangiocellular carcinoma (Topic) or intrahepatic cholangiocarcinoma (Topic) | 26819 |
| #2 | Radiomics (Topic) or Radiomic (Topic) or radiogenomic (Topic) or radiomics-based (Topic) or Texture (Topic) or machine learning (Topic) or Transfer Learning (Topic) or Deep learning (Topic) or Ensemble Learning (Topic) or artificial intelligence (Topic) or random forest (Topic) or neural network (Topic) or neural networks (Topic) or K-Nearest Neighbor (Topic) or CNN (Topic) or AlexNet (Topic) or VGGNet (Topic) or ResNet (Topic) or GoogLeNet (Topic) or Support vector machine (Topic) or SVM (Topic) or Gradient Boosting Machine (Topic) or Nomogram (Topic) or XGBoost (Topic) or Adaboost (Topic) or LightGBM (Topic) or CatBoost (Topic) or Decision tree (Topic) or Naive Bayesian (Topic) or Multilayer perceptron (Topic) or Bayesian network (Topic) or Prediction model (Topic) or Risk model (Topic) | 3632563 |
| #3 | #1 AND #2 | 1466 |
