# Supplementary material for: The Application Status of Radiomics-Based Machine Learning in Intrahepatic Cholangiocarcinoma: Systematic Review and Meta-Analysis
Source: J Med Internet Res. 2025 May 5;27:e69906. doi: 10.2196/69906 (PMC12089883; doi:10.2196/69906)
Supplement: Multimedia Appendix 2 [file jmir_v27i1e69906_app2.zip › Table S2.docx]

**Table S2** Characteristics of the included studies

| **Study ID** | **Country** | **Study type** | **Patient source** | **Outcome events** | **Radiomics source** | **Segmentation approach** | **Researchers** | **ROI region segmentation software** | **Number** | **Number_T** | **Generation method of validation set** | **Number_V** | **Model type** | **Manufacturer** | **Parameter** | **Variable screening method** |
| --- | --- | --- | --- | --- | --- | --- | --- | --- | --- | --- | --- | --- | --- | --- | --- | --- |
| Ying Xu(2024)[1] | China | Cohort study | Two centers | TLSs+RFS | MRI | Manual | 2 | ITKSNAP | 192 | 105 | Internal validation, external validation | set1:46 set2:41 | LR | General Electric Company, Boston, Massachusetts, USA | slice thickness/space: 5.0–8.0/1.0 mm, field-of-view: 36–42 cm, matrix size: 128 128, NEX: 4; | mRMR+LASSO |
| Luca Viganòa(2024)[2] | Italy | Case-control | Single center | Gene mutations | CT | Manual | 2 |  | 90 |  |  |  | LR |  |  | Multivariate LR |
| Li-Ya Su(2024)[3] | China | Case-control | Single center | Diagnosing ICC | US | Manual | 3 | Labelme | 280 | 224 | Random sampling | 56 | RF | Canon/Supersonic Explorer/Esaote/ Mindray | a frequency range of 1.0 to 6.0 MHz | XGBoost |
| Ziwei Liu(2024)[4] | China | Cohort study | Three centers | PNI | CT | Manual | 2 | 3D Slicer | 243 | 136 | Internal validation, external validation | set1:81 set2:26 | LR |  |  | Univariate +multivariate +ElasticNe |
| Francesco Fiz(2024)[5] | Italy | Cohort study | Six centers | OS+PFS | CT | Manual | 1 | LifeX | 215 |  |  |  | COX |  |  |  |
| Hüseyin Tuğsan Ballı(2024)[6] | Turkey | Case-control | Single center | Radioreactionof ICC on TARE | MRI | Manual | 2 | Olea Sphere | 36 |  |  |  | LR | Optima, General Electric Healthcare, USA | The parameters of axial T2W imaging were as follows: time of repetition (TR) 10,000 ms, time of echo (TE) 66 ms, layer thickness 6 mm, layer spacing 1 mm, matrix 320 × 320, field of view (FOV) 400 mm × 400 mm, piecewise collection times or average times 1, and parallel collection factor 0, fs. The parameters of dynamic CE MRI were as follows: TR 4.2 ms, TE 1 min full, layer thickness 5 mm, layer spacing 0 mm, matrix 260 × 224 mm, FOV 380 mm × 342 mm, and parallel acceleration factor 2. | LASSO |
| Ying Xu(2023)[7] | China | Case-control | Two centers | Diagnosing ICC | MRI | Manual | 3 | ITKSNAP | 241 | 133 | Internal validation, external validation | set1:57 set2:51 | LR |  |  | mRMR+LASSO |
| Ying Xu(2023)[8] | China | Cohort study | Two centers | TLSs+RFS | CT | Manual | 3 | ITKSNAP | 116 | 86 | External validation | 30 | LR |  |  | mRMR+LASSO |
| Yangda Song(2023)[9] | China | Case-control | Eight centers | Recurrence | CT | Manual | 3 | 3D Slicer | 311 |  | Internal validation, external validation | set1:36 set2:74 set3:61 | LightGBM |  |  | mRMR |
| Xianling Qian(2023)[10] | China | Case-control | Single center | Gene mutations | MRI | Manual | 2 | ITKSNAP | 173 | 124 | Random sampling, external validation | set1:54 set2:49 | LR+RF | United Imaging Healthcare, Shanghai, China |  | LASSO |
| S. Mahmoudi(2023)[11] | Germany | Case-control | Single center | Diagnosing ICC | CT | Manual | 3 | 3D Slicer | 94 | 65 | Random sampling | 29 | LR+ADB+SGB+RF | Somatom Force; Siemens Healthineers | rotation time, 0.5 s; collimation,0.6. | LASSO |
| Ning Liu(2023)[12] | China | Case-control | Single center | Diagnosing ICC | MRI | Manual | 1 | IBEX | 177 | 124 | Random sampling | 53 | LR | GE, USA |  | LASSO |
| Francesco Fiz(2023)[13] | Italy | Case-control | Six centers | G+MVI | CT | Manual | Multiple | LifeX | 244 |  |  |  | LR |  |  | Multivariate LR |
| Xiang Chen(2023)[14] | China | Case-control | Two centers | Gene mutations | MRI | Manual | 2 | MicroDicom | 78 | 53 | Random sampling | 25 | LR | Siemens Magnetom Verio 3.0 T; Siemens Magnetom Skyra 3.0 T; GE Signa HDxt 3.0 T |  |  |
| Xiang Chen(2023)[15] | China | Case-control | Single center | Diagnosing ICC | MRI | Manual | 2 | ITKSNAP | 134 | 93 | Random sampling | 41 | LR+RF+SGD+SVM | Magnetom Verio; Siemens Healthcare, Erlangen, Germany |  | LASSO |
| Shuang Chen(2023)[16] | China | Case-control | Single center | G+MVI | MRI | Manual | 2 | ITKSNAP | 235 | 167 | Random sampling | 68 | LR | GE Healthcare, Waukesha, WI, USA/Siemens, Erlangen, Germany |  | mRMR+LASSO |
| Zhiyuan Bo(2023)[17] | China | Case-control | Three centers | Recurrence | CT | Manual | 2 | MRIcroGL | 127 | 90 | Internal validation, external validation | 37 | LR+RF+NN+Bayes+SVM+LightGBM |  | tube voltage, 110–120 kVp; tube current, 130–375 mAs; rotation time,0.5–0.8 s; pixel spacing, 0.5–0.8 mm; slice thickness, 5 mm; image matrix, 512 × 512; and reconstruction interval, 5 mm. | mRMR |
| Yang Zhou(2022)[18] | China | Case-control | Single center | Diagnosing ICC | MRI | Manual | 2 | ITKSNAP | 216 | 151 | Internal validation, external validation | 65 | LR |  |  | LASSO |
| Shuaitong Zhang(2022)[19] | China | Cohort study | Two centers | OS+RFS+LN | CT | Manual | 2 | ITKSNAP | 296 | 243 | Internal validation, external validation | 53 | LR |  |  | mRMR |
| Yang Yang(2022)[20] | China | Cohort study | Single center | OS | MRI | Manual | 3 | 3D Slicer | 163 | 115 | Internal validation | 43 | COX | Discovery MR750, GE Medical Systems, Milwaukee, WI, USA; Magnetom Skyra, Siemens Medical Solutions, Erlangen, Germany |  | LASSO |
| Xiaoliang Xu(2022)[21] | China | Case-control | Single center | Diagnosing ICC | CT | Manual | 2 | 3D Slicer | 211 | 122 | Internal validation | 89 | SVM | LightSpeed, VCT, or Discovery HD 750, GE Healthcare, US | tube voltage 120 kVp, tube current 250–350mA, collimating slice thickness 5mm, reconstruction slice thickness 1.25mm, slice interval 5mm, rotation time 0.6 s, helical pitch 1.375, the field of view between 35 and 40 cm, and matrix 512 × 512. | LASSO |
| Xianling Qian(2022)[22] | China | Case-control | Two centers | MVI | MRI | Manual | 2 | ITKSNAP | 187 | 130 | Internal validation, external validation | set1:33 set2:24 | LR+RF+SVM | Siemens Healthcare, Erlangen, Germany |  | LASSO |
| Ming-De Li(2022)[23] | China | Cohort study | Single center | OS | US | Manual | 3 | ITKSNAP | 170 | 127 | Internal validation | 43 | LR | Toshiba Aplio 500, AlokaSSDa10, and SuperSonic Aixplorer |  | univariate and multivariate Cox regression |
| Joshua S. Jolissaint(2022)[24] | USA | Database | Single center | Recurrence | CT | Semi-automatic | 3 | Scout Liver | 138 | 97 | Internal validation | 41 | COX | LightSpeed 16-Slice and VCT, GE Healthcare, Boston, MA, USA | pitch/table speed 0.984–1.375/39.37–27.50 mm; autoMA 220–380; noise index 12.5–14; rotation time 0.7– 0.8 ms; scan delay 80–85 s. | MRMR |
| Chunjuan Jiang(2022)[25] | China | Case-control | Single center | Diagnosing ICC/MVI | PET/CT | Manual | 2 | 3D Slicer | 127/51 | 40 | Internal validation | 11 | RF | Siemens Healthiness, Knoxville, TN, USA | tube voltage, 120 kV; tube current, 80–250 mA; rotation time, 0.5 s; helical pitch 3.6; slice thickness, 5 mm; matrix, 512×512 | Wilcoxon test+RF+Sequential Forward Floating Algorithm |
| Feng Huang(2022)[26] | China | Case-control | Single center | Diagnosing ICC | MRI | Manual | 2 | ITKSNAP | 174 | 123 | Internal validation | 51 | LR |  | a TR of 9.1 ms, a TE of 1.33 ms, an echo spacing of 1.3 ms, the layer thickness of 5mm, an interval of 0mm, a FOV of 400mm× 350mm, and a matrix of 224 × 170. | mRMR+LASSO |
| Francesco Fiz(2022)[27] | Italy | Cohort study | Single center | G+MVI+OS+PFS | PET/CT | Manual | 2 | LifeX | 74 |  | Internal validation |  | LR+COX | Siemens, Erlangen, Germany |  | PCA |
| Jayasree Chakraborty(2022)[28] | USA | Database | Single center | Recurrence | CT | Semi-automatic | Multiple | Scout Liver | 139 |  |  |  | AdaBoost | Lightspeed 16 and VCT, GE Healthcare, Wisconsin | pitch/table speed = 0.984- 1.375/39.37-27.50 mm; autoMA 220-380; noise index 12.5-14; rotation time 0.7-0.8 ms; scan delay 80-85 s. | MRMR |
| Yong Zhu(2021)[29] | China | Cohort study | Single center | Gene mutations | CT | Manual | 2 | Pyradiomics | 138 |  |  |  | SVM | Lightspeed, VCT, or Discovery HD750, GE Healthcare, US |  | SVM |
| Yang Zhou(2021)[30] | China | Case-control | Single center | MVI | MRI | Manual | 2 | ITKSNAP | 126 | 88 | Internal validation | 38 | LASSO | United Imaging Healthcare |  | Pearson correlation analysis |
| Beihui Xue(2021)[31] | China | Case-control | Single center | Diagnosing ICC | CT | Manual | 2 | LifeX | 110 | 75 | Internal validation | 35 | LR |  |  | LASSO |
| Lei Xu(2021)[32] | China | Cohort study | Single center | Recurrence | MRI+CT | Manual | 2 | ITKSNAP | 209 | 159 | Internal validation | 50 | COX |  |  | MRMR |
| Hanyue Xu(2021)[33] | China | Case-control | Single center | Diagnosing ICC | CT | Manual | 3 | LifeX | 129 | 116 | Internal validation | 13 | RF+LDA | Philips Healthcare | 120 kVp, 200 mA, pitch 0.891 to 1.235; collimation 64 × 0.625 mm | DC+RF+LASSO+XGBoost+GBDT |
| Youyin Tang(2021)[34] | China | Cohort study | Single center | OS | CT | Manual | 2 | LifeX | 101 | 77 | Internal validation | 24 | COX | Brilliance 64, Philips Medical Systems, Eindhoven, the Netherlands | beam pitch, 0.891; tube voltage, 120 kVp; tube current, 200 mAs; detector collimation, 0.75 mm; slice thickness, 1.0 mm; reconstruction increment, 5.0 mm; rotation time, 0.42 s; and matrix, 512 × 512 | LASSO |
| Shanshan Ren(2021)[35] | China | Case-control | Three centers | Diagnosing ICC | US | Manual | 2 | ITKSNAP | 226 | 149 | Internal validation, external validation | set1:38 set2:39 | SVM |  | frequency range 2.5–6 MHz | LASSO |
| Xiaohan Hao(2021)[36] | China | Cohort study | Three centers | Recurrence | CT | Manual | 2 | ITKSNAP | 177 | 124 | Internal validation | 53 | MRMR-GBM | SOMATOM Definition AS+, Siemens Medical Solutions USA, Inc |  | MRMR |
| Hongpeng Chu(2021)[37] | China | Case-control | Two centers | Ineffective excision | CT | Manual | 3 | ITKSNAP | 203 | 142 | Internal validation | 61 | LR | Toshiba | 120 kV; 125 mAs; 0.5-s rotation time; 64 × 0.6 mm collimation; 60 × 360 mm fieldof-view; a pitch of 0.8; and a 512 × 512 matrix | RF |
| Jun Zhang(2020)[38] | China | Case-control | Single center | Diagnosing ICC | CT | Manual | 2 | ITKSNAP | 189 | 132 | Internal validation | 57 | COX | Philips Brilliance64, Philips Medical Systems, Best, The Netherlands; Siemens SOMATOM Definition Flash, Siemens Healthcare, Erlangen, Germany | voltage, 120 kV; current, 200–250 mAs; slice thickness, 5 mm | LASSO |
| Beihui Xue(2020)[39] | China | Case-control | Single center | Diagnosing ICC | CT | Manual | 2 | LifeX | 131 | 96 | External validation | 35 | LR |  |  | LASSO |
| Yu-ting Peng(2020)[40] | China | Case-control | Single center | MVI, PNI, differentiation, gene mutations, vascular endothelial growth factor and cytokeratin 7 | US | Manual | 2 | ITKSNAP | 128 |  | Internal validation |  | SVM+DT+LR+GBDT |  |  | LASSO+PCA |
| Yuting Peng(2020)[41] | China | Case-control | Single center | Diagnosing ICC | US | Manual | 2 | ITKSNAP | 137 | 95 | Internal validation | 42 | LR | GE Healthcare, United States, C5-1 abdominal probe, 2.8–5.0 MHz |  | LASSO+Spearman |
| Li Zhao(2019)[42] | China | Cohort study | Single center | Recurrence | MRI | Manual | 2 | ITKSNAP | 47 |  | Leave-one-out method |  | LR | Signa Excite HDxt, GE Healthcare, Milwaukee, USA |  | Univariate LR |
| Lei Xu(2019)[43] | China | Case-control | Single center | LN | MRI | Manual | 2 | ITKSNAP | 148 | 106 | External validation | 42 | SVM |  |  | MRMR |
| Sara Lewis(2019)[44] | USA | Case-control | Single center | Diagnosing ICC | MRI | Manual | 2 | Qsirix | 63 |  |  |  | LR | vanto and Aera, Siemens Healthineers; Signa HD, HDxt and Optima 450w, GE Medical Systems | FOV (238–420 × 339–480), matrix (80–192 × 108–160), slice thickness (6–9 mm), TR (3000–9200 ms), TE (54–84 ms), pixel bandwidth (1200–1953), and number of averages (1–8). | Wald criteria |
| Wenjie Liang(2018)[45] | China | Cohort study | Single center | Recurrence | MRI | Manual | 2 | ITKSNAP | 209 | 139 | External validation | 70 | LR | GE,Medical Systems, Milwaukee, WI, USA | repetition time (TR) of 2.8ms; echo time (TE) of 1.3ms; reverse time of 5ms; flip angle of 10◦; field of view of 380 × 304mm; bandwidth of 390.6 kHz; image resolution of 0.78 × 0.78 × 5mm. | Spearman’s rank correlation+LASSO |
| Ming-De Li(2024)[46] | China | Case-control | Single center | Diagnosing ICC | US |  | 3 |  | 1147 | 852 | Random sampling | 295 |  |  |  | ResNet-34+ConvLSTM+3D-CNN |
| Jianan Chen(2024)[47] | China | Case-control | Single center | Diagnosing ICC | US |  | 3 |  | 465 | 415 | Random sampling | 50 |  | Philips, GE, Canon, Esaote |  | Resnet18+MobileNet+DenseNet121+Inception V3 |
| Xuepeng Zhang(2023)[48] | China | Case-control | Single center | Diagnosing ICC | CT |  |  |  | 317 | 222 | Random sampling | 95 |  |  |  |  |
| Mengfan Xue(2023)[49] | China | Case-control | Single center | Diagnosing ICC | CT |  |  |  | 398 |  | Random sampling |  |  |  |  | Resnet18 |
| Abhishek Midya(2023)[50] | USA | Case-control | Three centers | Diagnosing ICC | CT | Semi-automatic | Multiple | Scout Liver | 814 |  | Random sampling | set1:162 set2:161 |  | Lightspeed 16 and VCT, GE Healthcare, Wisconsin | pitch/table speed = 0.984–1.375/39.37–27.50 mm; autoMA 220–380; noise index 12.5–14; rotation time 0.7–0.8 ms; and scan delay 80–85 s. The voxel size was [0.7324, 0.7324, 2.5] mm. | Inception v3 |
| Ji‑lan Huang(2023)[51] | China | Case-control | Single center | Diagnosing ICC | CT |  |  |  | 494 | 346 | Random sampling | set1:98 set2:50 |  | Siemens, Erlangen, Germany | voltage, 120 kV; current, 200–500 mA |  |
| Taiichi Wakiya(2022)[52] | Japan | Cohort study | Three centers | Recurrence | CT |  |  |  | 41 |  | 5-fold cross-validation |  |  |  |  | Resnet50 |
| Yangling Liu(2022)[53] | China | Case-control | Single center | Diagnosing ICC | MRI |  | 2 |  | 112 |  | Random sampling | set1:23 set2:25 |  | Sonata; Siemens Healthcare; Erlangen, Germany |  |  |
| Yating Ling(2022)[54] | China | Case-control | Single center | Diagnosing ICC | CT |  |  |  | 479 |  | Random sampling | 120 |  | Philips Healthcare, Netherlands |  | ResNet |
| Wenyu Gao(2022)[55] | China | Case-control | Three centers | MVI | MRI | Manual | 3 | ITKS  NAP | 519 |  | Random sampling ,external validation | set1:90 set2:68 |  |  |  |  |
| QIYUAN WANG(2020)[56] | China | Case-control | Single center | Diagnosing ICC | CT | Semi-automatic | Multiple |  | 234 |  | Random sampling | 307 |  |  |  |  |
| Donlapark Ponnoprat(2020)[57] | Thailand | Case-control | Two centers | Diagnosing ICC | CT | Automatic |  |  | 257 | 157 | 10-fold cross-validation | 17 |  | SOMATOM definition, Siemens, Germany | 120 kVp, 200–400 mAs, 0.6 mm × 64 section collimation with a single breath-hold helical acquisition. | U-Net |
| Abhishek Midyaa(2018)[58] | USA | Case-control | Single center | Diagnosing ICC | CT | Semi-automatic | Multiple | Scout Liver | 223 | 156 | Random sampling | 67 |  |  |  | Inception v3 |

Note：(1)Number-the number of cases included in each study; Number_T-the number of cases in the training set in each study; Number_V-the number of cases in the validation set in each study

USA: United States of America; ICC: intrahepatic cholangiocarcinoma; CT: computed tomography; MRI: magnetic resonance imaging; PNI: perineural invasion; MVI:microvascular invasion; TLSs: tertiary lymphoid structures; OS: overall survival; LN: lymph node; PFS: progression-free survival; RFS: recurrence free survival; TARE: transarterial radioembolization; G: tumor grade; LR: logistic regression; SVM: support vector machine; SGD: stochastic gradient descent; SGB: stochastic gradient boosting; DT: decision tree; GBDT: gradient boosting decision tree ;ADB: AdaBoost, adaptive boosting; LASSO:least absolute shrinkage and selection operator; LightGBM:light gradient boosting machine; MRMR-GBM: minimum redundancy maximum relevance gradient boosting machine; RF: random forest; NN: neural network; LDA: linear discriminant analysis; COX: cox proportional hazards.

**References**

1. Xu Y, Li Z, Yang Y, Zhang YW, Li L, Zhou YZ, et al. Association Between MRI Radiomics and Intratumoral Tertiary Lymphoid Structures in Intrahepatic Cholangiocarcinoma and Its Prognostic Significance. Journal of Magnetic Resonance Imaging. 2024 Aug;60(2):715-28. PMID: WOS:001102554900001. doi: 10.1002/jmri.29128.

2. Viganò L, Zanuso V, Fiz F, Cerri L, Laino ME, Ammirabile A, et al. CT-based radiogenomics of intrahepatic cholangiocarcinoma. Digestive and Liver Disease. 2024. doi: 10.1016/j.dld.2024.06.033.

3. Su LY, Xu M, Chen YL, Lin MX, Xie XY. Ultrasomics in liver cancer: Developing a radiomics model for differentiating intrahepatic cholangiocarcinoma from hepatocellular carcinoma using contrast-enhanced ultrasound. World Journal of Radiology. 2024 Jul;16(7). PMID: WOS:001281105400004. doi: 10.4329/wjr.v16.i7.247.

4. Liu ZW, Luo C, Chen XJ, Feng YQ, Feng JY, Zhang R, et al. Noninvasive prediction of perineural invasion in intrahepatic cholangiocarcinoma by clinicoradiological features and computed tomography radiomics based on interpretable machine learning: a multicenter cohort study. International Journal of Surgery. 2024 Feb;110(2):1039-51. PMID: WOS:001164676800039. doi: 10.1097/js9.0000000000000881.

5. Fiz F, Rossi N, Langella S, Conci S, Serenari M, Ardito F, et al. Radiomics of Intrahepatic Cholangiocarcinoma and Peritumoral Tissue Predicts Postoperative Survival: Development of a CT-Based Clinical-Radiomic Model. Annals of surgical oncology. 2024 Sep;31(9):5604-14. PMID: 38797789. doi: 10.1245/s10434-024-15457-9.

6. Ballı HT, Pişkin FC, Püren Yücel S, Sözütok S, Özgül D, Aikimbaev K. Predictability of the radiological response to Yttrium-90 transarterial radioembolization by dynamic magnetic resonance imaging-based radiomics analysis in patients with intrahepatic cholangiocarcinoma. Diagnostic and interventional radiology (Ankara, Turkey). 2024 May 13;30(3):193-9. PMID: 36994655. doi: 10.4274/dir.2023.222025.

7. Xu Y, Ye F, Li L, Yang Y, Ouyang J, Zhou Y, et al. MRI-Based Radiomics Nomogram for Preoperatively Differentiating Intrahepatic Mass-Forming Cholangiocarcinoma From Resectable Colorectal Liver Metastases. Academic radiology. 2023 Sep;30(9):2010-20. PMID: 37414635. doi: 10.1016/j.acra.2023.04.030.

8. Xu Y, Li Z, Yang Y, Li L, Zhou Y, Ouyang J, et al. A CT-based radiomics approach to predict intra-tumoral tertiary lymphoid structures and recurrence of intrahepatic cholangiocarcinoma. Insights into Imaging. 2023;14(1). doi: 10.1186/s13244-023-01527-1.

9. Song Y, Zhou G, Zhou Y, Xu Y, Zhang J, Zhang K, et al. Artificial intelligence CT radiomics to predict early recurrence of intrahepatic cholangiocarcinoma: a multicenter study. Hepatology international. 2023 Aug;17(4):1016-27. PMID: 36821045. doi: 10.1007/s12072-023-10487-z.

10. Qian X, Zhou C, Wang F, Lu X, Zhang Y, Chen L, et al. Development and validation of combined Ki67 status prediction model for intrahepatic cholangiocarcinoma based on clinicoradiological features and MRI radiomics. La Radiologia medica. 2023 Mar;128(3):274-88. PMID: 36773271. doi: 10.1007/s11547-023-01597-7.

11. Mahmoudi S, Bernatz S, Ackermann J, Koch V, Dos Santos DP, Grünewald LD, et al. Computed Tomography Radiomics to Differentiate Intrahepatic Cholangiocarcinoma and Hepatocellular Carcinoma. Clinical oncology (Royal College of Radiologists (Great Britain)). 2023 May;35(5):e312-e8. PMID: 36804153. doi: 10.1016/j.clon.2023.01.018.

12. Liu N, Wu Y, Tao Y, Zheng J, Huang X, Yang L, et al. Differentiation of Hepatocellular Carcinoma from Intrahepatic Cholangiocarcinoma through MRI Radiomics. Cancers. 2023;15(22). doi: 10.3390/cancers15225373.

13. Fiz F, Rossi N, Langella S, Ruzzenente A, Serenari M, Ardito F, et al. Radiomic Analysis of Intrahepatic Cholangiocarcinoma: Non-Invasive Prediction of Pathology Data: A Multicenter Study to Develop a Clinical-Radiomic Model. Cancers. 2023 Sep;15(17). PMID: WOS:001070056800001. doi: 10.3390/cancers15174204.

14. Chen X, Zhu J, Zou Z, Du M, Xie J, Ye Y, et al. Nomogram based on MRI for preoperative prediction of Ki-67 expression in patients with intrahepatic mass cholangiocarcinoma. Abdominal radiology (New York). 2023 Feb;48(2):567-78. PMID: 36401626. doi: 10.1007/s00261-022-03719-7.

15. Chen X, Chen Y, Chen H, Zhu J, Huang R, Xie J, et al. Machine learning based on gadoxetic acid-enhanced MRI for differentiating atypical intrahepatic mass-forming cholangiocarcinoma from poorly differentiated hepatocellular carcinoma. Abdominal radiology (New York). 2023 Aug;48(8):2525-36. PMID: 37169988. doi: 10.1007/s00261-023-03870-9.

16. Chen S, Zhu YM, Wan LJ, Zou SM, Zhang HM. Predicting the microvascular invasion and tumor grading of intrahepatic mass-forming cholangiocarcinoma based on magnetic resonance imaging radiomics and morphological features. Quantitative Imaging in Medicine and Surgery. 2023 Dec;13(12):8079-+. PMID: WOS:001087770800001. doi: 10.21037/qims-23-11.

17. Bo Z, Chen B, Yang Y, Zhao Z, Wang Y, Chen G. Machine learning radiomics to predict the early recurrence of intrahepatic cholangiocarcinoma after curative resection: a multicenter cohort study. Cancer Research. 2023;83(7). doi: DOI: 10.1007/s00259-023-06184-6.

18. Zhou Y, Zhou G, Zhang J, Xu C, Zhu F, Xu P. DCE-MRI based radiomics nomogram for preoperatively differentiating combined hepatocellular-cholangiocarcinoma from mass-forming intrahepatic cholangiocarcinoma. European radiology. 2022 Jul;32(7):5004-15. PMID: 35128572. doi: 10.1007/s00330-022-08548-2.

19. Zhang S, Huang S, He W, Wei J, Huo L, Jia N, et al. Radiomics-Based Preoperative Prediction of Lymph Node Metastasis in Intrahepatic Cholangiocarcinoma Using Contrast-Enhanced Computed Tomography. Annals of surgical oncology. 2022 Oct;29(11):6786-99. PMID: 35789309. doi: 10.1245/s10434-022-12028-8.

20. Yang Y, Zou X, Zhou W, Yuan G, Hu D, Kuang D, et al. Multiparametric MRI-Based Radiomic Signature for Preoperative Evaluation of Overall Survival in Intrahepatic Cholangiocarcinoma After Partial Hepatectomy. Journal of magnetic resonance imaging : JMRI. 2022 Sep;56(3):739-51. PMID: 35049076. doi: 10.1002/jmri.28071.

21. Xu X, Mao Y, Tang Y, Liu Y, Xue C, Yue Q, et al. Classification of Hepatocellular Carcinoma and Intrahepatic Cholangiocarcinoma Based on Radiomic Analysis. Computational and mathematical methods in medicine. 2022;2022:5334095. PMID: 35237341. doi: 10.1155/2022/5334095.

22. Qian X, Lu X, Ma X, Zhang Y, Zhou C, Wang F, et al. A Multi-Parametric Radiomics Nomogram for Preoperative Prediction of Microvascular Invasion Status in Intrahepatic Cholangiocarcinoma. Frontiers in Oncology. 2022;12. doi: 10.3389/fonc.2022.838701.

23. Li MD, Lu XZ, Liu JF, Chen B, Xu M, Xie XY, et al. Preoperative Survival Prediction in Intrahepatic Cholangiocarcinoma Using a Ultrasound-Based Radiographic-Radiomics Signature. Journal of Ultrasound in Medicine. 2022 Jun;41(6):1483-95. PMID: WOS:000697617800001. doi: 10.1002/jum.15833.

24. Jolissaint JS, Wang T, Soares KC, Chou JF, Gönen M, Pak LM, et al. Machine learning radiomics can predict early liver recurrence after resection of intrahepatic cholangiocarcinoma. HPB : the official journal of the International Hepato Pancreato Biliary Association. 2022 Aug;24(8):1341-50. PMID: 35283010. doi: 10.1016/j.hpb.2022.02.004.

25. Jiang C, Zhao L, Xin B, Ma G, Wang X, Song S. 18F-FDG PET/CT radiomic analysis for classifying and predicting microvascular invasion in hepatocellular carcinoma and intrahepatic cholangiocarcinoma. Quantitative Imaging in Medicine and Surgery. 2022;12(8):4135-50. doi: 10.21037/qims-21-1167.

26. Huang F, Liu X, Liu P, Xu D, Li Z, Lin H, et al. The Application Value of MRI T2(∗)WI Radiomics Nomogram in Discriminating Hepatocellular Carcinoma from Intrahepatic Cholangiocarcinoma. Computational and mathematical methods in medicine. 2022;2022:7099476. PMID: 36203532. doi: 10.1155/2022/7099476.

27. Fiz F, Masci C, Costa G, Sollini M, Chiti A, Ieva F, et al. PET/CT-based radiomics of mass-forming intrahepatic cholangiocarcinoma improves prediction of pathology data and survival. European journal of nuclear medicine and molecular imaging. 2022 Aug;49(10):3387-400. PMID: 35347437. doi: 10.1007/s00259-022-05765-1.

28. Chakraborty J, Jolissaint JS, Wang TG, Soares KC, Gönen M, Pak LM, et al., editors. CT Radiomics to Predict Early Hepatic Recurrence after Resection for Intrahepatic Cholangiocarcinoma. Conference on Medical Imaging - Computer-Aided Diagnosis; 2022 Feb 20-Mar 27; Electr Network; 2022.

29. Zhu Y, Mao YF, Chen J, Qiu YD, Guan Y, Wang ZQ, et al. Value of contrast-enhanced CT texture analysis in predicting IDH mutation status of intrahepatic cholangiocarcinoma. Scientific Reports. 2021 Mar;11(1). PMID: WOS:000635702100016. doi: 10.1038/s41598-021-86497-4.

30. Zhou Y, Zhou G, Zhang J, Xu C, Wang X, Xu P. Radiomics signature on dynamic contrast-enhanced MR images: a potential imaging biomarker for prediction of microvascular invasion in mass-forming intrahepatic cholangiocarcinoma. European radiology. 2021 Sep;31(9):6846-55. PMID: 33638019. doi: 10.1007/s00330-021-07793-1.

31. Xue B, Wu S, Zhang M, Hong J, Liu B, Xu N, et al. A radiomic-based model of different contrast-enhanced CT phase for differentiate intrahepatic cholangiocarcinoma from inflammatory mass with hepatolithiasis. Abdominal radiology (New York). 2021 Aug;46(8):3835-44. PMID: 33728532. doi: 10.1007/s00261-021-03027-6.

32. Xu L, Wan Y, Luo C, Yang J, Yang P, Chen F, et al. Integrating intratumoral and peritumoral features to predict tumor recurrence in intrahepatic cholangiocarcinoma. Physics in medicine and biology. 2021 Jun 7;66(12). PMID: 34096890. doi: 10.1088/1361-6560/ac01f3.

33. Xu H, Zou X, Zhao Y, Zhang T, Tang Y, Zheng A, et al. Differentiation of Intrahepatic Cholangiocarcinoma and Hepatic Lymphoma Based on Radiomics and Machine Learning in Contrast-Enhanced Computer Tomography. Technology in cancer research & treatment. 2021 Jan-Dec;20:15330338211039125. PMID: 34499018. doi: 10.1177/15330338211039125.

34. Tang Y, Zhang T, Zhou X, Zhao Y, Xu H, Liu Y, et al. The preoperative prognostic value of the radiomics nomogram based on CT combined with machine learning in patients with intrahepatic cholangiocarcinoma. World journal of surgical oncology. 2021 Aug 1;19(1):45. PMID: 34334138. doi: 10.1186/s12957-021-02162-0.

35. Ren S, Li Q, Liu S, Qi Q, Duan S, Mao B, et al. Clinical Value of Machine Learning-Based Ultrasomics in Preoperative Differentiation Between Hepatocellular Carcinoma and Intrahepatic Cholangiocarcinoma: A Multicenter Study. Frontiers in Oncology. 2021;11. doi: 10.3389/fonc.2021.749137.

36. Hao X, Liu B, Hu X, Wei J, Han Y, Liu X, et al. A Radiomics-based Approach for Predicting Early Recurrence in Intrahepatic Cholangiocarcinoma after Surgical Resection: A Multicenter Study. Annual International Conference of the IEEE Engineering in Medicine and Biology Society IEEE Engineering in Medicine and Biology Society Annual International Conference. 2021 Nov;2021:3659-62. PMID: 34892030. doi: 10.1109/embc46164.2021.9630029.

37. Chu H, Liu Z, Liang W, Zhou Q, Zhang Y, Lei K, et al. Radiomics using CT images for preoperative prediction of futile resection in intrahepatic cholangiocarcinoma. European radiology. 2021 Apr;31(4):2368-76. PMID: 33033863. doi: 10.1007/s00330-020-07250-5.

38. Zhang J, Huang Z, Cao L, Zhang Z, Wei Y, Zhang X, et al. Differentiation combined hepatocellular and cholangiocarcinoma from intrahepatic cholangiocarcinoma based on radiomics machine learning. Annals of Translational Medicine. 2020;8(4). doi: 10.21037/atm.2020.01.126.

39. Xue B, Wu S, Zheng M, Jiang H, Chen J, Jiang Z, et al. Development and Validation of a Radiomic-Based Model for Prediction of Intrahepatic Cholangiocarcinoma in Patients With Intrahepatic Lithiasis Complicated by Imagologically Diagnosed Mass. Frontiers in Oncology. 2020;10. doi: 10.3389/fonc.2020.598253.

40. Peng YT, Zhou CY, Lin P, Wen DY, Wang XD, Zhong XZ, et al. Preoperative Ultrasound Radiomics Signatures for Noninvasive Evaluation of Biological Characteristics of Intrahepatic Cholangiocarcinoma. Academic radiology. 2020 Jun;27(6):785-97. PMID: 31494003. doi: 10.1016/j.acra.2019.07.029.

41. Peng Y, Lin P, Wu L, Wan D, Zhao Y, Liang L, et al. Ultrasound-Based Radiomics Analysis for Preoperatively Predicting Different Histopathological Subtypes of Primary Liver Cancer. Frontiers in Oncology. 2020;10. doi: 10.3389/fonc.2020.01646.

42. Zhao L, Ma X, Liang M, Li D, Ma P, Wang S, et al. Prediction for early recurrence of intrahepatic mass-forming cholangiocarcinoma: quantitative magnetic resonance imaging combined with prognostic immunohistochemical markers. Cancer imaging : the official publication of the International Cancer Imaging Society. 2019 Jul 15;19(1):49. PMID: 31307551. doi: 10.1186/s40644-019-0234-4.

43. Xu L, Yang P, Liang W, Liu W, Wang W, Luo C, et al. A radiomics approach based on support vector machine using MR images for preoperative lymph node status evaluation in intrahepatic cholangiocarcinoma. Theranostics. 2019;9(18):5374-85. PMID: 31410221. doi: 10.7150/thno.34149.

44. Lewis S, Peti S, Hectors SJ, King M, Rosen A, Kamath A, et al. Volumetric quantitative histogram analysis using diffusion-weighted magnetic resonance imaging to differentiate HCC from other primary liver cancers. Abdominal Radiology. 2019;44(3):912-22. doi: 10.1007/s00261-019-01906-7.

45. Liang W, Xu L, Yang P, Zhang L, Wan D, Huang Q, et al. Novel nomogram for preoperative prediction of early recurrence in intrahepatic cholangiocarcinoma. Frontiers in Oncology. 2018;8(SEP). doi: 10.3389/fonc.2018.00360.

46. Li MD, Li W, Lin MX, Lin XX, Hu HT, Wang YC, et al. Systematic comparison of deep-learning based fusion strategies for multi-modal ultrasound in diagnosis of liver cancer. Neurocomputing. 2024;603. doi: 10.1016/j.neucom.2024.128257.

47. Chen J, Zhang W, Bao J, Wang K, Zhao Q, Zhu Y, et al. Implications of ultrasound-based deep learning model for preoperatively differentiating combined hepatocellular-cholangiocarcinoma from hepatocellular carcinoma and intrahepatic cholangiocarcinoma. Abdominal radiology (New York). 2024 Jan;49(1):93-102. PMID: 37999743. doi: 10.1007/s00261-023-04089-4.

48. Zhang X, Jia N, Wang Y. Multi-input dense convolutional network for classification of hepatocellular carcinoma and intrahepatic cholangiocarcinoma. Biomedical Signal Processing and Control. 2023;80. doi: 10.1016/j.bspc.2022.104226.

49. Xue M, Jiang H, Zheng J, Wu Y, Xu Y, Pan J, et al. Spatiotemporal Excitation Module-based CNN for Diagnosis of Hepatic Malignancy in Four-phase CT Images. Annual International Conference of the IEEE Engineering in Medicine and Biology Society IEEE Engineering in Medicine and Biology Society Annual International Conference. 2023;2023:1-5. doi: 10.1109/EMBC40787.2023.10340787.

50. Midya A, Chakraborty J, Srouji R, Narayan RR, Boerner T, Zheng J, et al. Computerized Diagnosis of Liver Tumors From CT Scans Using a Deep Neural Network Approach. IEEE journal of biomedical and health informatics. 2023 May;27(5):2456-64. PMID: 37027632. doi: 10.1109/jbhi.2023.3248489.

51. Huang JL, Sun Y, Wu ZH, Zhu HJ, Xia GJ, Zhu XS, et al. Differential diagnosis of hepatocellular carcinoma and intrahepatic cholangiocarcinoma based on spatial and channel attention mechanisms. Journal of cancer research and clinical oncology. 2023 Sep;149(12):10161-8. PMID: 37268850. doi: 10.1007/s00432-023-04935-4.

52. Wakiya T, Ishido K, Kimura N, Nagase H, Kanda T, Ichiyama S, et al. CT-based deep learning enables early postoperative recurrence prediction for intrahepatic cholangiocarcinoma. Sci Rep. 2022 May 19;12(1):8428. PMID: 35590089. doi: 10.1038/s41598-022-12604-8.

53. Liu Y, Wang B, Mo X, Tang K, He J, Hao J. A Deep Learning Workflow for Mass-Forming Intrahepatic Cholangiocarcinoma and Hepatocellular Carcinoma Classification Based on MRI. Current oncology (Toronto, Ont). 2022 Dec 30;30(1):529-44. PMID: 36661691. doi: 10.3390/curroncol30010042.

54. Ling Y, Ying S, Xu L, Peng Z, Mao X, Chen Z, et al. Automatic volumetric diagnosis of hepatocellular carcinoma based on four-phase CT scans with minimum extra information. Frontiers in Oncology. 2022;12. doi: 10.3389/fonc.2022.960178.

55. Gao W, Wang W, Song D, Wang K, Lian D, Yang C, et al. A Multiparametric Fusion Deep Learning Model Based on DCE-MRI for Preoperative Prediction of Microvascular Invasion in Intrahepatic Cholangiocarcinoma. Journal of magnetic resonance imaging : JMRI. 2022 Oct;56(4):1029-39. PMID: 35191550. doi: 10.1002/jmri.28126.

56. Wang QY, Wang ZM, Sun Y, Zhang X, Li WF, Ge Y, et al. SCCNN: A Diagnosis Method for Hepatocellular Carcinoma and Intrahepatic Cholangiocarcinoma Based on Siamese Cross Contrast Neural Network. Ieee Access. 2020;8:85271-83. PMID: WOS:000549851400001. doi: 10.1109/access.2020.2992627.

57. Ponnoprat D, Inkeaw P, Chaijaruwanich J, Traisathit P, Sripan P, Inmutto N, et al. Classification of hepatocellular carcinoma and intrahepatic cholangiocarcinoma based on multi-phase CT scans. Medical & Biological Engineering & Computing. 2020 Oct;58(10):2497-515. PMID: WOS:000559498900001. doi: 10.1007/s11517-020-02229-2.

58. Midya A, Chakraborty J, Pak LM, Zheng J, Jarnagin WR, Do RKG, et al., editors. Deep Convolutional Neural Network for the Classification of Hepatocellular Carcinoma and Intrahepatic Cholangiocarcinoma. Conference on Medical Imaging - Computer-Aided Diagnosis; 2018 Feb 12-15; Houston, TX; 2018.
