# Supplementary material for: The Application Status of Radiomics-Based Machine Learning in Intrahepatic Cholangiocarcinoma: Systematic Review and Meta-Analysis
Source: J Med Internet Res. 2025 May 5;27:e69906. doi: 10.2196/69906 (PMC12089883; doi:10.2196/69906)
Supplement: Multimedia Appendix 2 [file jmir_v27i1e69906_app2.zip › Table S3.docx]

**Table S3** RQS assessment results for the included studies

| **Study ID** | **v1** | **v2** | **v3** | **v4** | **v5** | **v6** | **v7** | **v8** | **v9** | **v10** | **v11** | **v12** | **v13** | **v14** | **v15** | **v16** | **Total** | **Proportion** |
| --- | --- | --- | --- | --- | --- | --- | --- | --- | --- | --- | --- | --- | --- | --- | --- | --- | --- | --- |
| Ying Xu(2024)[1] | 1 | 1 | 0 | 0 | 0 | 0 | 0 | 1 | 1 | 0 | 0 | 4 | 0 | 2 | 0 | 1 | 11 | 30.56 |
| Luca Viganòa(2024)[2] | 0 | 1 | 0 | 0 | 0 | 1 | 0 | 0 | 1 | 1 | 0 | -5 | 0 | 0 | 0 | 1 | 0 | 0.00 |
| Li-Ya Su(2024)[3] | 1 | 1 | 0 | 1 | 0 | 1 | 0 | 0 | 1 | 0 | 0 | 2 | 0 | 2 | 0 | 1 | 10 | 27.78 |
| Ziwei Liu(2024)[4] | 1 | 1 | 0 | 1 | 0 | 1 | 0 | 0 | 1 | 0 | 0 | 4 | 0 | 0 | 0 | 1 | 10 | 27.78 |
| Francesco Fiz(2024)[5] | 0 | 0 | 0 | 1 | 0 | 1 | 0 | 0 | 1 | 0 | 0 | -5 | 0 | 0 | 0 | 0 | -2 | -5.56 |
| Hüseyin Tuğsan Ballı(2024)[6] | 1 | 1 | 0 | 1 | 0 | 1 | 0 | 1 | 1 | 0 | 0 | -5 | 0 | 0 | 0 | 1 | 2 | 5.56 |
| Ying Xu(2023)[7] | 1 | 1 | 0 | 1 | 0 | 1 | 0 | 0 | 1 | 0 | 0 | 4 | 0 | 0 | 0 | 1 | 10 | 27.78 |
| Ying Xu(2023)[8] | 1 | 1 | 0 | 1 | 0 | 1 | 0 | 1 | 1 | 0 | 0 | 3 | 2 | 2 | 0 | 1 | 14 | 38.89 |
| Yangda Song(2023)[9] | 1 | 1 | 0 | 1 | 0 | 1 | 0 | 0 | 1 | 1 | 0 | 5 | 2 | 0 | 0 | 1 | 14 | 38.89 |
| Xianling Qian(2023)[10] | 1 | 1 | 0 | 1 | 0 | 1 | 0 | 0 | 1 | 1 | 0 | 2 | 0 | 0 | 1 | 1 | 10 | 27.78 |
| S. Mahmoudi(2023)[11] | 1 | 1 | 0 | 0 | 0 | 1 | 0 | 1 | 1 | 1 | 0 | 2 | 0 | 0 | 0 | 1 | 9 | 25.00 |
| Ning Liu(2023)[12] | 1 | 0 | 0 | 1 | 0 | 1 | 0 | 1 | 1 | 0 | 0 | 2 | 0 | 0 | 0 | 1 | 8 | 22.22 |
| Francesco Fiz(2023)[13] | 0 | 1 | 0 | 1 | 0 | 1 | 0 | 0 | 2 | 1 | 0 | 5 | 0 | 0 | 0 | 1 | 12 | 33.33 |
| Xiang Chen(2023)[14] | 1 | 1 | 0 | 1 | 0 | 1 | 0 | 0 | 1 | 1 | 0 | 3 | 0 | 2 | 0 | 1 | 12 | 33.33 |
| Xiang Chen(2023)[15] | 1 | 1 | 0 | 1 | 0 | 1 | 0 | 0 | 1 | 0 | 0 | 2 | 0 | 2 | 0 | 1 | 10 | 27.78 |
| Shuang Chen(2023)[16] | 1 | 1 | 0 | 1 | 0 | 1 | 0 | 1 | 1 | 1 | 0 | 2 | 0 | 2 | 0 | 1 | 12 | 33.33 |
| Zhiyuan Bo(2023)[17] | 1 | 1 | 0 | 0 | 0 | 1 | 0 | 0 | 1 | 1 | 0 | 5 | 0 | 2 | 0 | 1 | 13 | 36.11 |
| Yang Zhou(2022)[18] | 1 | 1 | 0 | 1 | 0 | 1 | 0 | 1 | 1 | 1 | 0 | 2 | 0 | 0 | 0 | 1 | 10 | 27.78 |
| Shuaitong Zhang(2022)[19] | 1 | 1 | 0 | 1 | 0 | 1 | 0 | 0 | 1 | 1 | 0 | 4 | 0 | 2 | 0 | 1 | 13 | 36.11 |
| Yang Yang(2022)[20] | 1 | 1 | 0 | 1 | 0 | 1 | 0 | 1 | 2 | 1 | 0 | 2 | 0 | 0 | 0 | 1 | 11 | 30.56 |
| Xiaoliang Xu(2022)[21] | 1 | 1 | 0 | 0 | 0 | 1 | 0 | 0 | 1 | 1 | 0 | 2 | 0 | 2 | 0 | 0 | 9 | 25.00 |
| Xianling Qian(2022)[22] | 1 | 1 | 0 | 1 | 0 | 0 | 0 | 1 | 1 | 1 | 0 | 4 | 0 | 2 | 1 | 1 | 14 | 38.89 |
| Ming-De Li(2022)[23] | 1 | 1 | 0 | 1 | 0 | 1 | 0 | 0 | 1 | 1 | 0 | 2 | 2 | 0 | 0 | 1 | 11 | 30.56 |
| Joshua S. Jolissaint(2022)[24] | 1 | 1 | 0 | 0 | 0 | 1 | 0 | 0 | 1 | 0 | 0 | 2 | 0 | 0 | 0 | 1 | 7 | 19.44 |
| Chunjuan Jiang(2022)[25] | 1 | 1 | 0 | 0 | 0 | 1 | 0 | 0 | 1 | 0 | 0 | 2 | 0 | 0 | 0 | 1 | 7 | 19.44 |
| Feng Huang(2022)[26] | 1 | 1 | 0 | 0 | 0 | 1 | 0 | 1 | 2 | 2 | 0 | 2 | 0 | 0 | 0 | 1 | 11 | 30.56 |
| Francesco Fiz(2022)[27] | 1 | 1 | 0 | 0 | 0 | 1 | 0 | 0 | 2 | 1 | 0 | 2 | 0 | 0 | 0 | 0 | 8 | 22.22 |
| Jayasree Chakraborty(2022)[28] | 1 | 1 | 0 | 0 | 0 | 0 | 0 | 1 | 2 | 1 | 0 | 2 | 0 | 0 | 0 | 1 | 9 | 25.00 |
| Yong Zhu(2021)[29] | 1 | 1 | 0 | 1 | 0 | 0 | 0 | 0 | 2 | 1 | 0 | 2 | 0 | 0 | 0 | 1 | 9 | 25.00 |
| Yang Zhou(2021)[30] | 1 | 1 | 0 | 1 | 0 | 1 | 0 | 1 | 2 | 1 | 0 | 2 | 0 | 0 | 0 | 1 | 11 | 30.56 |
| Beihui Xue(2021)[31] | 1 | 1 | 0 | 1 | 0 | 1 | 0 | 0 | 2 | 1 | 0 | 2 | 0 | 2 | 0 | 1 | 12 | 33.33 |
| Lei Xu(2021)[32] | 1 | 1 | 0 | 1 | 0 | 1 | 0 | 1 | 2 | 1 | 0 | 2 | 0 | 0 | 0 | 1 | 11 | 30.56 |
| Hanyue Xu(2021)[33] | 1 | 1 | 0 | 1 | 0 | 1 | 0 | 0 | 2 | 1 | 0 | 2 | 0 | 0 | 0 | 1 | 10 | 27.78 |
| Youyin Tang(2021)[34] | 1 | 1 | 0 | 1 | 0 | 1 | 0 | 1 | 1 | 1 | 0 | 2 | 0 | 2 | 0 | 1 | 12 | 33.33 |
| Shanshan Ren(2021)[35] | 1 | 1 | 0 | 0 | 0 | 1 | 0 | 0 | 2 | 1 | 0 | 4 | 0 | 0 | 0 | 1 | 11 | 30.56 |
| Xiaohan Hao(2021)[36] | 1 | 1 | 0 | 0 | 0 | 1 | 0 | 0 | 2 | 1 | 0 | 5 | 0 | 0 | 0 | 1 | 12 | 33.33 |
| Hongpeng Chu(2021)[37] | 1 | 1 | 0 | 1 | 0 | 1 | 0 | 0 | 1 | 0 | 0 | 4 | 0 | 0 | 0 | 1 | 10 | 27.78 |
| Jun Zhang(2020)[38] | 1 | 1 | 0 | 1 | 0 | 1 | 0 | 1 | 1 | 0 | 0 | 2 | 0 | 2 | 0 | 1 | 11 | 30.56 |
| Beihui Xue(2020)[39] | 1 | 1 | 0 | 0 | 0 | 1 | 0 | 1 | 1 | 1 | 0 | 2 | 0 | 2 | 0 | 1 | 11 | 30.56 |
| Yu-ting Peng(2020)[40] | 1 | 1 | 0 | 0 | 0 | 1 | 0 | 0 | 1 | 0 | 0 | 2 | 0 | 0 | 0 | 1 | 7 | 19.44 |
| Yuting Peng(2020)[41] | 1 | 1 | 0 | 0 | 0 | 1 | 0 | 1 | 1 | 0 | 0 | 2 | 0 | 0 | 0 | 1 | 8 | 22.22 |
| Li Zhao(2019)[42] | 1 | 1 | 0 | 1 | 0 | 1 | 0 | 0 | 1 | 0 | 0 | 2 | 0 | 0 | 0 | 1 | 8 | 22.22 |
| Lei Xu(2019)[43] | 1 | 1 | 0 | 0 | 0 | 1 | 0 | 1 | 1 | 2 | 0 | 2 | 0 | 2 | 0 | 1 | 12 | 33.33 |
| Sara Lewis(2019)[44] | 1 | 1 | 0 | 1 | 0 | 1 | 0 | 0 | 1 | 0 | 0 | -5 | 0 | 0 | 0 | 1 | 1 | 2.78 |
| Wenjie Liang(2018)[45] | 1 | 1 | 0 | 1 | 0 | 1 | 0 | 1 | 1 | 1 | 0 | 2 | 0 | 2 | 0 | 1 | 12 | 33.33 |
| Ming-De Li(2024)[46] | 0 | 1 | 0 | 1 | 0 | 1 | 0 | 0 | 1 | 0 | 0 | 2 | 0 | 0 | 0 | 1 | 7 | 19.44 |
| Jianan Chen(2024)[47] | 0 | 1 | 0 | 0 | 0 | 1 | 0 | 0 | 2 | 1 | 0 | 2 | 0 | 0 | 0 | 1 | 8 | 22.22 |
| Xuepeng Zhang(2023)[48] | 0 | 1 | 0 | 1 | 0 | 0 | 0 | 0 | 1 | 0 | 0 | 2 | 0 | 0 | 0 | 1 | 6 | 16.67 |
| Mengfan Xue(2023)[49] | 0 | 1 | 0 | 1 | 0 | 0 | 0 | 0 | 2 | 1 | 0 | 2 | 0 | 0 | 0 | 1 | 8 | 22.22 |
| Abhishek Midya(2023)[50] | 1 | 1 | 0 | 0 | 0 | 0 | 0 | 0 | 1 | 0 | 0 | 4 | 0 | 0 | 0 | 1 | 8 | 22.22 |
| Ji‑lan Huang(2023)[51] | 1 | 1 | 0 | 1 | 0 | 0 | 0 | 0 | 2 | 1 | 0 | 2 | 0 | 2 | 0 | 0 | 10 | 27.78 |
| Taiichi Wakiya(2022)[52] | 0 | 1 | 0 | 0 | 0 | 1 | 0 | 0 | 2 | 1 | 0 | 5 | 0 | 0 | 0 | 1 | 11 | 30.56 |
| Yangling Liu(2022)[53] | 1 | 1 | 0 | 0 | 0 | 0 | 0 | 0 | 1 | 0 | 0 | 2 | 0 | 0 | 0 | 0 | 5 | 13.89 |
| Yating Ling(2022)[54] | 1 | 1 | 0 | 0 | 0 | 0 | 0 | 0 | 2 | 1 | 0 | 2 | 0 | 0 | 0 | 1 | 8 | 22.22 |
| Wenyu Gao(2022)[55] | 1 | 1 | 0 | 1 | 0 | 0 | 0 | 0 | 1 | 0 | 0 | 4 | 0 | 0 | 0 | 1 | 9 | 25.00 |
| QIYUAN WANG(2020)[56] | 1 | 1 | 0 | 1 | 0 | 0 | 0 | 0 | 1 | 0 | 0 | 2 | 0 | 0 | 0 | 1 | 7 | 19.44 |
| Donlapark Ponnoprat(2020)[57] | 1 | 1 | 0 | 1 | 0 | 0 | 0 | 0 | 2 | 1 | 0 | 2 | 0 | 0 | 0 | 1 | 9 | 25.00 |
| Abhishek Midyaa(2018)[58] | 0 | 1 | 0 | 0 | 0 | 0 | 0 | 0 | 1 | 0 | 0 | 2 | 0 | 0 | 0 | 1 | 5 | 13.89 |

Note:

v1-Image protocol:+ 1 if protocols are well-documented + 1 if public protocol is used

V2-Multiple segmentations:+ 1 segmentation by different physicians/algorithms/software or at different breathing cycles/perturbing segmentations by (random) noise

V3-Phantom study:+1 if inter-scanner differences and vendor-dependent features were used for feature robustness assessment

V4-Multiple Time Points: +1 multiple time points for feature robustness assessment

V5-Feature Reduction: - 3 if neither measure is implemented + 3 if either measure is implemented

V6-Non Radiomics: +1 if multivariable analysis with non-radiomics features

V7-Biological Correlates: +1 if phenotypic differences are demonstrated

V8-Cut-off: +1 if risk groups determined by either the median, a previously published cut-off or report a continuous risk variable

V9-Discrimination statistics: +1 if a discrimination statistic and its statistical significance are reported + 1 if a resampling method technique is also applied

V10-Calibration statistics: +1 if a calibration statistic and its statistical significance are reported + 1 if a resampling method technique is also applied

V11-Prospective study registration: + 7 for prospective validation of a radiomics signature in an appropriate trial

V12-Validation: - 5 if validation is missing/+ 2 if validation is based on a dataset from the same institute/+ 3 if validation is based on a dataset from another institute/+ 4 if validation is based on two datasets from two distinct institutes/+ 4 if the study validates a previously published signature/+ 5 if validation is based on three or more datasets from distinct institutes

V13-Comparison to ‘gold standard’: +2 assess the extent to which the model agrees with/is superior to the current ‘gold standard’ method

V14-Potential clinical utility: +2 if the potential clinical utility reported

V15-Cost-effectiveness: +1 if the cost-effectiveness of the clinical application is reported

V16-Open science and data: + 1 if scans are open source/+ 1 if region of interest segmentations are open source/+ 1 if code is open source/+ 1 if radiomics features are calculated on a set of representative ROIs and the calculated features and representative ROIs are open source

**References**

1. Xu Y, Li Z, Yang Y, Zhang YW, Li L, Zhou YZ, et al. Association Between MRI Radiomics and Intratumoral Tertiary Lymphoid Structures in Intrahepatic Cholangiocarcinoma and Its Prognostic Significance. Journal of Magnetic Resonance Imaging. 2024 Aug;60(2):715-28. PMID: WOS:001102554900001. doi: 10.1002/jmri.29128.

2. Viganò L, Zanuso V, Fiz F, Cerri L, Laino ME, Ammirabile A, et al. CT-based radiogenomics of intrahepatic cholangiocarcinoma. Digestive and Liver Disease. 2024. doi: 10.1016/j.dld.2024.06.033.

3. Su LY, Xu M, Chen YL, Lin MX, Xie XY. Ultrasomics in liver cancer: Developing a radiomics model for differentiating intrahepatic cholangiocarcinoma from hepatocellular carcinoma using contrast-enhanced ultrasound. World Journal of Radiology. 2024 Jul;16(7). PMID: WOS:001281105400004. doi: 10.4329/wjr.v16.i7.247.

4. Liu ZW, Luo C, Chen XJ, Feng YQ, Feng JY, Zhang R, et al. Noninvasive prediction of perineural invasion in intrahepatic cholangiocarcinoma by clinicoradiological features and computed tomography radiomics based on interpretable machine learning: a multicenter cohort study. International Journal of Surgery. 2024 Feb;110(2):1039-51. PMID: WOS:001164676800039. doi: 10.1097/js9.0000000000000881.

5. Fiz F, Rossi N, Langella S, Conci S, Serenari M, Ardito F, et al. Radiomics of Intrahepatic Cholangiocarcinoma and Peritumoral Tissue Predicts Postoperative Survival: Development of a CT-Based Clinical-Radiomic Model. Annals of surgical oncology. 2024 Sep;31(9):5604-14. PMID: 38797789. doi: 10.1245/s10434-024-15457-9.

6. Ballı HT, Pişkin FC, Püren Yücel S, Sözütok S, Özgül D, Aikimbaev K. Predictability of the radiological response to Yttrium-90 transarterial radioembolization by dynamic magnetic resonance imaging-based radiomics analysis in patients with intrahepatic cholangiocarcinoma. Diagnostic and interventional radiology (Ankara, Turkey). 2024 May 13;30(3):193-9. PMID: 36994655. doi: 10.4274/dir.2023.222025.

7. Xu Y, Ye F, Li L, Yang Y, Ouyang J, Zhou Y, et al. MRI-Based Radiomics Nomogram for Preoperatively Differentiating Intrahepatic Mass-Forming Cholangiocarcinoma From Resectable Colorectal Liver Metastases. Academic radiology. 2023 Sep;30(9):2010-20. PMID: 37414635. doi: 10.1016/j.acra.2023.04.030.

8. Xu Y, Li Z, Yang Y, Li L, Zhou Y, Ouyang J, et al. A CT-based radiomics approach to predict intra-tumoral tertiary lymphoid structures and recurrence of intrahepatic cholangiocarcinoma. Insights into Imaging. 2023;14(1). doi: 10.1186/s13244-023-01527-1.

9. Song Y, Zhou G, Zhou Y, Xu Y, Zhang J, Zhang K, et al. Artificial intelligence CT radiomics to predict early recurrence of intrahepatic cholangiocarcinoma: a multicenter study. Hepatology international. 2023 Aug;17(4):1016-27. PMID: 36821045. doi: 10.1007/s12072-023-10487-z.

10. Qian X, Zhou C, Wang F, Lu X, Zhang Y, Chen L, et al. Development and validation of combined Ki67 status prediction model for intrahepatic cholangiocarcinoma based on clinicoradiological features and MRI radiomics. La Radiologia medica. 2023 Mar;128(3):274-88. PMID: 36773271. doi: 10.1007/s11547-023-01597-7.

11. Mahmoudi S, Bernatz S, Ackermann J, Koch V, Dos Santos DP, Grünewald LD, et al. Computed Tomography Radiomics to Differentiate Intrahepatic Cholangiocarcinoma and Hepatocellular Carcinoma. Clinical oncology (Royal College of Radiologists (Great Britain)). 2023 May;35(5):e312-e8. PMID: 36804153. doi: 10.1016/j.clon.2023.01.018.

12. Liu N, Wu Y, Tao Y, Zheng J, Huang X, Yang L, et al. Differentiation of Hepatocellular Carcinoma from Intrahepatic Cholangiocarcinoma through MRI Radiomics. Cancers. 2023;15(22). doi: 10.3390/cancers15225373.

13. Fiz F, Rossi N, Langella S, Ruzzenente A, Serenari M, Ardito F, et al. Radiomic Analysis of Intrahepatic Cholangiocarcinoma: Non-Invasive Prediction of Pathology Data: A Multicenter Study to Develop a Clinical-Radiomic Model. Cancers. 2023 Sep;15(17). PMID: WOS:001070056800001. doi: 10.3390/cancers15174204.

14. Chen X, Zhu J, Zou Z, Du M, Xie J, Ye Y, et al. Nomogram based on MRI for preoperative prediction of Ki-67 expression in patients with intrahepatic mass cholangiocarcinoma. Abdominal radiology (New York). 2023 Feb;48(2):567-78. PMID: 36401626. doi: 10.1007/s00261-022-03719-7.

15. Chen X, Chen Y, Chen H, Zhu J, Huang R, Xie J, et al. Machine learning based on gadoxetic acid-enhanced MRI for differentiating atypical intrahepatic mass-forming cholangiocarcinoma from poorly differentiated hepatocellular carcinoma. Abdominal radiology (New York). 2023 Aug;48(8):2525-36. PMID: 37169988. doi: 10.1007/s00261-023-03870-9.

16. Chen S, Zhu YM, Wan LJ, Zou SM, Zhang HM. Predicting the microvascular invasion and tumor grading of intrahepatic mass-forming cholangiocarcinoma based on magnetic resonance imaging radiomics and morphological features. Quantitative Imaging in Medicine and Surgery. 2023 Dec;13(12):8079-+. PMID: WOS:001087770800001. doi: 10.21037/qims-23-11.

17. Bo Z, Chen B, Yang Y, Zhao Z, Wang Y, Chen G. Machine learning radiomics to predict the early recurrence of intrahepatic cholangiocarcinoma after curative resection: a multicenter cohort study. Cancer Research. 2023;83(7). doi: DOI: 10.1007/s00259-023-06184-6.

18. Zhou Y, Zhou G, Zhang J, Xu C, Zhu F, Xu P. DCE-MRI based radiomics nomogram for preoperatively differentiating combined hepatocellular-cholangiocarcinoma from mass-forming intrahepatic cholangiocarcinoma. European radiology. 2022 Jul;32(7):5004-15. PMID: 35128572. doi: 10.1007/s00330-022-08548-2.

19. Zhang S, Huang S, He W, Wei J, Huo L, Jia N, et al. Radiomics-Based Preoperative Prediction of Lymph Node Metastasis in Intrahepatic Cholangiocarcinoma Using Contrast-Enhanced Computed Tomography. Annals of surgical oncology. 2022 Oct;29(11):6786-99. PMID: 35789309. doi: 10.1245/s10434-022-12028-8.

20. Yang Y, Zou X, Zhou W, Yuan G, Hu D, Kuang D, et al. Multiparametric MRI-Based Radiomic Signature for Preoperative Evaluation of Overall Survival in Intrahepatic Cholangiocarcinoma After Partial Hepatectomy. Journal of magnetic resonance imaging : JMRI. 2022 Sep;56(3):739-51. PMID: 35049076. doi: 10.1002/jmri.28071.

21. Xu X, Mao Y, Tang Y, Liu Y, Xue C, Yue Q, et al. Classification of Hepatocellular Carcinoma and Intrahepatic Cholangiocarcinoma Based on Radiomic Analysis. Computational and mathematical methods in medicine. 2022;2022:5334095. PMID: 35237341. doi: 10.1155/2022/5334095.

22. Qian X, Lu X, Ma X, Zhang Y, Zhou C, Wang F, et al. A Multi-Parametric Radiomics Nomogram for Preoperative Prediction of Microvascular Invasion Status in Intrahepatic Cholangiocarcinoma. Frontiers in Oncology. 2022;12. doi: 10.3389/fonc.2022.838701.

23. Li MD, Lu XZ, Liu JF, Chen B, Xu M, Xie XY, et al. Preoperative Survival Prediction in Intrahepatic Cholangiocarcinoma Using a Ultrasound-Based Radiographic-Radiomics Signature. Journal of Ultrasound in Medicine. 2022 Jun;41(6):1483-95. PMID: WOS:000697617800001. doi: 10.1002/jum.15833.

24. Jolissaint JS, Wang T, Soares KC, Chou JF, Gönen M, Pak LM, et al. Machine learning radiomics can predict early liver recurrence after resection of intrahepatic cholangiocarcinoma. HPB : the official journal of the International Hepato Pancreato Biliary Association. 2022 Aug;24(8):1341-50. PMID: 35283010. doi: 10.1016/j.hpb.2022.02.004.

25. Jiang C, Zhao L, Xin B, Ma G, Wang X, Song S. 18F-FDG PET/CT radiomic analysis for classifying and predicting microvascular invasion in hepatocellular carcinoma and intrahepatic cholangiocarcinoma. Quantitative Imaging in Medicine and Surgery. 2022;12(8):4135-50. doi: 10.21037/qims-21-1167.

26. Huang F, Liu X, Liu P, Xu D, Li Z, Lin H, et al. The Application Value of MRI T2(∗)WI Radiomics Nomogram in Discriminating Hepatocellular Carcinoma from Intrahepatic Cholangiocarcinoma. Computational and mathematical methods in medicine. 2022;2022:7099476. PMID: 36203532. doi: 10.1155/2022/7099476.

27. Fiz F, Masci C, Costa G, Sollini M, Chiti A, Ieva F, et al. PET/CT-based radiomics of mass-forming intrahepatic cholangiocarcinoma improves prediction of pathology data and survival. European journal of nuclear medicine and molecular imaging. 2022 Aug;49(10):3387-400. PMID: 35347437. doi: 10.1007/s00259-022-05765-1.

28. Chakraborty J, Jolissaint JS, Wang TG, Soares KC, Gönen M, Pak LM, et al., editors. CT Radiomics to Predict Early Hepatic Recurrence after Resection for Intrahepatic Cholangiocarcinoma. Conference on Medical Imaging - Computer-Aided Diagnosis; 2022 Feb 20-Mar 27; Electr Network; 2022.

29. Zhu Y, Mao YF, Chen J, Qiu YD, Guan Y, Wang ZQ, et al. Value of contrast-enhanced CT texture analysis in predicting IDH mutation status of intrahepatic cholangiocarcinoma. Scientific Reports. 2021 Mar;11(1). PMID: WOS:000635702100016. doi: 10.1038/s41598-021-86497-4.

30. Zhou Y, Zhou G, Zhang J, Xu C, Wang X, Xu P. Radiomics signature on dynamic contrast-enhanced MR images: a potential imaging biomarker for prediction of microvascular invasion in mass-forming intrahepatic cholangiocarcinoma. European radiology. 2021 Sep;31(9):6846-55. PMID: 33638019. doi: 10.1007/s00330-021-07793-1.

31. Xue B, Wu S, Zhang M, Hong J, Liu B, Xu N, et al. A radiomic-based model of different contrast-enhanced CT phase for differentiate intrahepatic cholangiocarcinoma from inflammatory mass with hepatolithiasis. Abdominal radiology (New York). 2021 Aug;46(8):3835-44. PMID: 33728532. doi: 10.1007/s00261-021-03027-6.

32. Xu L, Wan Y, Luo C, Yang J, Yang P, Chen F, et al. Integrating intratumoral and peritumoral features to predict tumor recurrence in intrahepatic cholangiocarcinoma. Physics in medicine and biology. 2021 Jun 7;66(12). PMID: 34096890. doi: 10.1088/1361-6560/ac01f3.

33. Xu H, Zou X, Zhao Y, Zhang T, Tang Y, Zheng A, et al. Differentiation of Intrahepatic Cholangiocarcinoma and Hepatic Lymphoma Based on Radiomics and Machine Learning in Contrast-Enhanced Computer Tomography. Technology in cancer research & treatment. 2021 Jan-Dec;20:15330338211039125. PMID: 34499018. doi: 10.1177/15330338211039125.

34. Tang Y, Zhang T, Zhou X, Zhao Y, Xu H, Liu Y, et al. The preoperative prognostic value of the radiomics nomogram based on CT combined with machine learning in patients with intrahepatic cholangiocarcinoma. World journal of surgical oncology. 2021 Aug 1;19(1):45. PMID: 34334138. doi: 10.1186/s12957-021-02162-0.

35. Ren S, Li Q, Liu S, Qi Q, Duan S, Mao B, et al. Clinical Value of Machine Learning-Based Ultrasomics in Preoperative Differentiation Between Hepatocellular Carcinoma and Intrahepatic Cholangiocarcinoma: A Multicenter Study. Frontiers in Oncology. 2021;11. doi: 10.3389/fonc.2021.749137.

36. Hao X, Liu B, Hu X, Wei J, Han Y, Liu X, et al. A Radiomics-based Approach for Predicting Early Recurrence in Intrahepatic Cholangiocarcinoma after Surgical Resection: A Multicenter Study. Annual International Conference of the IEEE Engineering in Medicine and Biology Society IEEE Engineering in Medicine and Biology Society Annual International Conference. 2021 Nov;2021:3659-62. PMID: 34892030. doi: 10.1109/embc46164.2021.9630029.

37. Chu H, Liu Z, Liang W, Zhou Q, Zhang Y, Lei K, et al. Radiomics using CT images for preoperative prediction of futile resection in intrahepatic cholangiocarcinoma. European radiology. 2021 Apr;31(4):2368-76. PMID: 33033863. doi: 10.1007/s00330-020-07250-5.

38. Zhang J, Huang Z, Cao L, Zhang Z, Wei Y, Zhang X, et al. Differentiation combined hepatocellular and cholangiocarcinoma from intrahepatic cholangiocarcinoma based on radiomics machine learning. Annals of Translational Medicine. 2020;8(4). doi: 10.21037/atm.2020.01.126.

39. Xue B, Wu S, Zheng M, Jiang H, Chen J, Jiang Z, et al. Development and Validation of a Radiomic-Based Model for Prediction of Intrahepatic Cholangiocarcinoma in Patients With Intrahepatic Lithiasis Complicated by Imagologically Diagnosed Mass. Frontiers in Oncology. 2020;10. doi: 10.3389/fonc.2020.598253.

40. Peng YT, Zhou CY, Lin P, Wen DY, Wang XD, Zhong XZ, et al. Preoperative Ultrasound Radiomics Signatures for Noninvasive Evaluation of Biological Characteristics of Intrahepatic Cholangiocarcinoma. Academic radiology. 2020 Jun;27(6):785-97. PMID: 31494003. doi: 10.1016/j.acra.2019.07.029.

41. Peng Y, Lin P, Wu L, Wan D, Zhao Y, Liang L, et al. Ultrasound-Based Radiomics Analysis for Preoperatively Predicting Different Histopathological Subtypes of Primary Liver Cancer. Frontiers in Oncology. 2020;10. doi: 10.3389/fonc.2020.01646.

42. Zhao L, Ma X, Liang M, Li D, Ma P, Wang S, et al. Prediction for early recurrence of intrahepatic mass-forming cholangiocarcinoma: quantitative magnetic resonance imaging combined with prognostic immunohistochemical markers. Cancer imaging : the official publication of the International Cancer Imaging Society. 2019 Jul 15;19(1):49. PMID: 31307551. doi: 10.1186/s40644-019-0234-4.

43. Xu L, Yang P, Liang W, Liu W, Wang W, Luo C, et al. A radiomics approach based on support vector machine using MR images for preoperative lymph node status evaluation in intrahepatic cholangiocarcinoma. Theranostics. 2019;9(18):5374-85. PMID: 31410221. doi: 10.7150/thno.34149.

44. Lewis S, Peti S, Hectors SJ, King M, Rosen A, Kamath A, et al. Volumetric quantitative histogram analysis using diffusion-weighted magnetic resonance imaging to differentiate HCC from other primary liver cancers. Abdominal Radiology. 2019;44(3):912-22. doi: 10.1007/s00261-019-01906-7.

45. Liang W, Xu L, Yang P, Zhang L, Wan D, Huang Q, et al. Novel nomogram for preoperative prediction of early recurrence in intrahepatic cholangiocarcinoma. Frontiers in Oncology. 2018;8(SEP). doi: 10.3389/fonc.2018.00360.

46. Li MD, Li W, Lin MX, Lin XX, Hu HT, Wang YC, et al. Systematic comparison of deep-learning based fusion strategies for multi-modal ultrasound in diagnosis of liver cancer. Neurocomputing. 2024;603. doi: 10.1016/j.neucom.2024.128257.

47. Chen J, Zhang W, Bao J, Wang K, Zhao Q, Zhu Y, et al. Implications of ultrasound-based deep learning model for preoperatively differentiating combined hepatocellular-cholangiocarcinoma from hepatocellular carcinoma and intrahepatic cholangiocarcinoma. Abdominal radiology (New York). 2024 Jan;49(1):93-102. PMID: 37999743. doi: 10.1007/s00261-023-04089-4.

48. Zhang X, Jia N, Wang Y. Multi-input dense convolutional network for classification of hepatocellular carcinoma and intrahepatic cholangiocarcinoma. Biomedical Signal Processing and Control. 2023;80. doi: 10.1016/j.bspc.2022.104226.

49. Xue M, Jiang H, Zheng J, Wu Y, Xu Y, Pan J, et al. Spatiotemporal Excitation Module-based CNN for Diagnosis of Hepatic Malignancy in Four-phase CT Images. Annual International Conference of the IEEE Engineering in Medicine and Biology Society IEEE Engineering in Medicine and Biology Society Annual International Conference. 2023;2023:1-5. doi: 10.1109/EMBC40787.2023.10340787.

50. Midya A, Chakraborty J, Srouji R, Narayan RR, Boerner T, Zheng J, et al. Computerized Diagnosis of Liver Tumors From CT Scans Using a Deep Neural Network Approach. IEEE journal of biomedical and health informatics. 2023 May;27(5):2456-64. PMID: 37027632. doi: 10.1109/jbhi.2023.3248489.

51. Huang JL, Sun Y, Wu ZH, Zhu HJ, Xia GJ, Zhu XS, et al. Differential diagnosis of hepatocellular carcinoma and intrahepatic cholangiocarcinoma based on spatial and channel attention mechanisms. Journal of cancer research and clinical oncology. 2023 Sep;149(12):10161-8. PMID: 37268850. doi: 10.1007/s00432-023-04935-4.

52. Wakiya T, Ishido K, Kimura N, Nagase H, Kanda T, Ichiyama S, et al. CT-based deep learning enables early postoperative recurrence prediction for intrahepatic cholangiocarcinoma. Sci Rep. 2022 May 19;12(1):8428. PMID: 35590089. doi: 10.1038/s41598-022-12604-8.

53. Liu Y, Wang B, Mo X, Tang K, He J, Hao J. A Deep Learning Workflow for Mass-Forming Intrahepatic Cholangiocarcinoma and Hepatocellular Carcinoma Classification Based on MRI. Current oncology (Toronto, Ont). 2022 Dec 30;30(1):529-44. PMID: 36661691. doi: 10.3390/curroncol30010042.

54. Ling Y, Ying S, Xu L, Peng Z, Mao X, Chen Z, et al. Automatic volumetric diagnosis of hepatocellular carcinoma based on four-phase CT scans with minimum extra information. Frontiers in Oncology. 2022;12. doi: 10.3389/fonc.2022.960178.

55. Gao W, Wang W, Song D, Wang K, Lian D, Yang C, et al. A Multiparametric Fusion Deep Learning Model Based on DCE-MRI for Preoperative Prediction of Microvascular Invasion in Intrahepatic Cholangiocarcinoma. Journal of magnetic resonance imaging : JMRI. 2022 Oct;56(4):1029-39. PMID: 35191550. doi: 10.1002/jmri.28126.

56. Wang QY, Wang ZM, Sun Y, Zhang X, Li WF, Ge Y, et al. SCCNN: A Diagnosis Method for Hepatocellular Carcinoma and Intrahepatic Cholangiocarcinoma Based on Siamese Cross Contrast Neural Network. Ieee Access. 2020;8:85271-83. PMID: WOS:000549851400001. doi: 10.1109/access.2020.2992627.

57. Ponnoprat D, Inkeaw P, Chaijaruwanich J, Traisathit P, Sripan P, Inmutto N, et al. Classification of hepatocellular carcinoma and intrahepatic cholangiocarcinoma based on multi-phase CT scans. Medical & Biological Engineering & Computing. 2020 Oct;58(10):2497-515. PMID: WOS:000559498900001. doi: 10.1007/s11517-020-02229-2.

58. Midya A, Chakraborty J, Pak LM, Zheng J, Jarnagin WR, Do RKG, et al., editors. Deep Convolutional Neural Network for the Classification of Hepatocellular Carcinoma and Intrahepatic Cholangiocarcinoma. Conference on Medical Imaging - Computer-Aided Diagnosis; 2018 Feb 12-15; Houston, TX; 2018.
